# Novel Vascular‐Adaptive Liquid Metal Microspheres Enable Visualized Arterial Embolization Therapy

**DOI:** 10.1002/advs.202521441

**Published:** 2026-03-20

**Authors:** Chenyu Shen, Junge Chen, Gang Zhang, Zhusheng Liu, Yichen Wan, Lei Lei, Weiyan Ren, Yanyu Zhao, Bozhang Xia, Zhiqiang Yi, Jinjin Wang, Jingwang Gao, Gen Mu, Xing‐Jie Liang, Zhijun Wang

**Affiliations:** ^1^ CAS Key Laboratory for Biomedical Effects of Nanomaterials and Nanosafety CAS Center for Excellence in Nanoscience National Center for Nanoscience and Technology of China Beijing China; ^2^ Department of Geriatric Medicine & National Clinical Research Centre of Geriatric Disease The Second Medical Center Department of Interventional Radiology Chinese PLA General Hospital Beijing China; ^3^ Tangshan Gongren Hospital Tangshan Hebei China; ^4^ School of Engineering Medicine Beijing Advanced Innovation Center for Biomedical Engineering School of Integrated Circuit Science and Engineering Beihang University Beijing China; ^5^ Department of Neurosurgery Peking University First Hospital Beijing China; ^6^ Bioengineering University of Pennsylvania Philadelphia Pennsylvania USA

**Keywords:** arterial embolization therapy, drug delivery, liquid metal, microsphere, visualization

## Abstract

Arterial embolization therapy is a promising strategy for treating both malignant and benign tumors. However, conventional embolic agents often lack inherent radiopacity and have limited embolizing ability, resulting in difficulty in real‐time monitoring during arterial embolization, low postoperative tumor necrosis rate, and high risk of recurrence. In this study, we prepared a liquid metal microsphere with radiopacity by disrupting the surface tension of liquid gallium via ultrasonication. These microspheres have a self‐limiting oxide layer on their surface, while their core remains liquid. Drug loading can be achieved by modifying the surface of liquid metal microspheres, and the drug‐loaded microspheres are named X‐MEN. Owing to their unique physical structure, these liquid metal microspheres exhibit excellent fluidity, viscoelasticity, and deformability. This enables them to navigate through microcatheters and conform tightly to the vessel wall, achieving efficient embolization. These microspheres can remain stable in the target vessel for at least six months, with no observed recanalization. In addition, the radiopacity of these microspheres allows for real‐time monitoring during arterial embolization, thereby enabling precise control over the embolization process. Therefore, liquid metal microspheres are a very promising long‐acting embolic agent for image‐guided arterial embolization.

## Graphical abstract

We present drug‐loaded liquid metal microspheres (X‐MEN) as a novel agent for image‐guided arterial embolization. With inherent radiopacity and superior deformability, X‐MEN enables real‐time monitoring and conforms tightly to irregular vessels. This approach ensures precise, long‐lasting embolization without recanalization, addressing critical clinical challenges in treating both malignant and benign tumors.

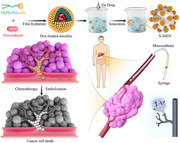

## 1 Introduction

Arterial Embolization therapy is a commonly used therapeutic strategy for tumors, in which embolic agents are injected into the blood supply arteries of the tumor through a catheter to block the supply of oxygen and nutrients to the tumor to starve the tumor of nutrients [1, 2, 3]. The success of embolization is largely dependent on the types of embolic agents used. As a representative of liquid embolic agents, iodized oil can be deposited in tumors and blood vessels and shows radiopacity and drug‐carrying capacity [4, 5]. However, the embolic capability of iodized oil is limited, and it is prone to causing vascular recanalization [6]. As a novel solid embolic agent, microspheres can be readily suspended in liquid, allowing smooth passage through catheters, and exhibit enhanced embolization efficacy and biocompatibility [7, 8, 9]. Microspheres designed with different materials exhibit varying embolization efficacy. During the embolization process, microspheres with enhanced elasticity are more likely to pass through catheters and reach smaller blood vessels [10, 11, 12]. Structurally engineered Janus‐Lipiodol droplets with viscoelastic deformation capabilities, designed by Tao et al., achieved efficient embolization in rabbit kidneys [13]. Embolization using microspheres with different size distributions in target vessels of varying diameters also results in improved embolization efficacy [14, 15, 16]. However, due to the relatively large size range of these microspheres (e.g., 100–300 µm) and their disordered distribution, larger diameter microspheres may preferentially embolize proximal vessels, leading to insufficient embolization of the microvasculature [3, 17]. Additionally, the radiopacity of microspheres is a crucial feature, enabling real‐time monitoring of microsphere positioning during embolization and post‐procedural observation of microsphere distribution. However, most embolic microspheres used clinically lack inherent radiopacity. Therefore, there is a pressing need to develop a novel embolic material that enables visualization and complete occlusion of the arterial embolization process, thereby enhancing the therapeutic efficacy of the procedure.

Gallium (Ga) is a metal that is liquid at or near room temperature and is used in a wide range of applications [18, 19, 20, 21, 22, 23, 24, 25, 26, 27]. In previous work, we found that liquid metal particles can be generated via disrupting the surface tension of the gallium‐based liquid metal, which is stabilized by a functionalized or oxide layer [28, 29, 30, 31]. Gallium‐based liquid metal particles have good biocompatibility, especially when they are modified with biocompatible polymers, such as polyethylene glycol‐sulfhydryl [32, 33, 34]. In recent years, gallium‐based liquid metal particles have been explored for the treatment of oncological diseases, including drug delivery [35, 36, 37, 38], potential utility as intravascular embolic agents [37], and photothermal or magnetothermal treatment of tumors [39, 40]. In our previous work, we found that gallium‐based liquid metal particles could be used to enhance cryotherapy of tumors [30, 41]. In addition, Ga‐based liquid metal particles are used in bioimaging to achieve x‐ray visualization of bolus agents by dispersing them in hydrogels or adsorbing them to bolus microspheres to provide radiopacity [41, 42]. Micelles are a highly promising platform that can serve as carriers for poorly water‐soluble chemotherapeutic drugs [43, 44, 45]. Encapsulation of chemotherapeutic agents in micelles improves pharmacokinetic profiles and increases therapeutic efficacy while reducing side effects during treatment [46].

**SCHEME 1:**
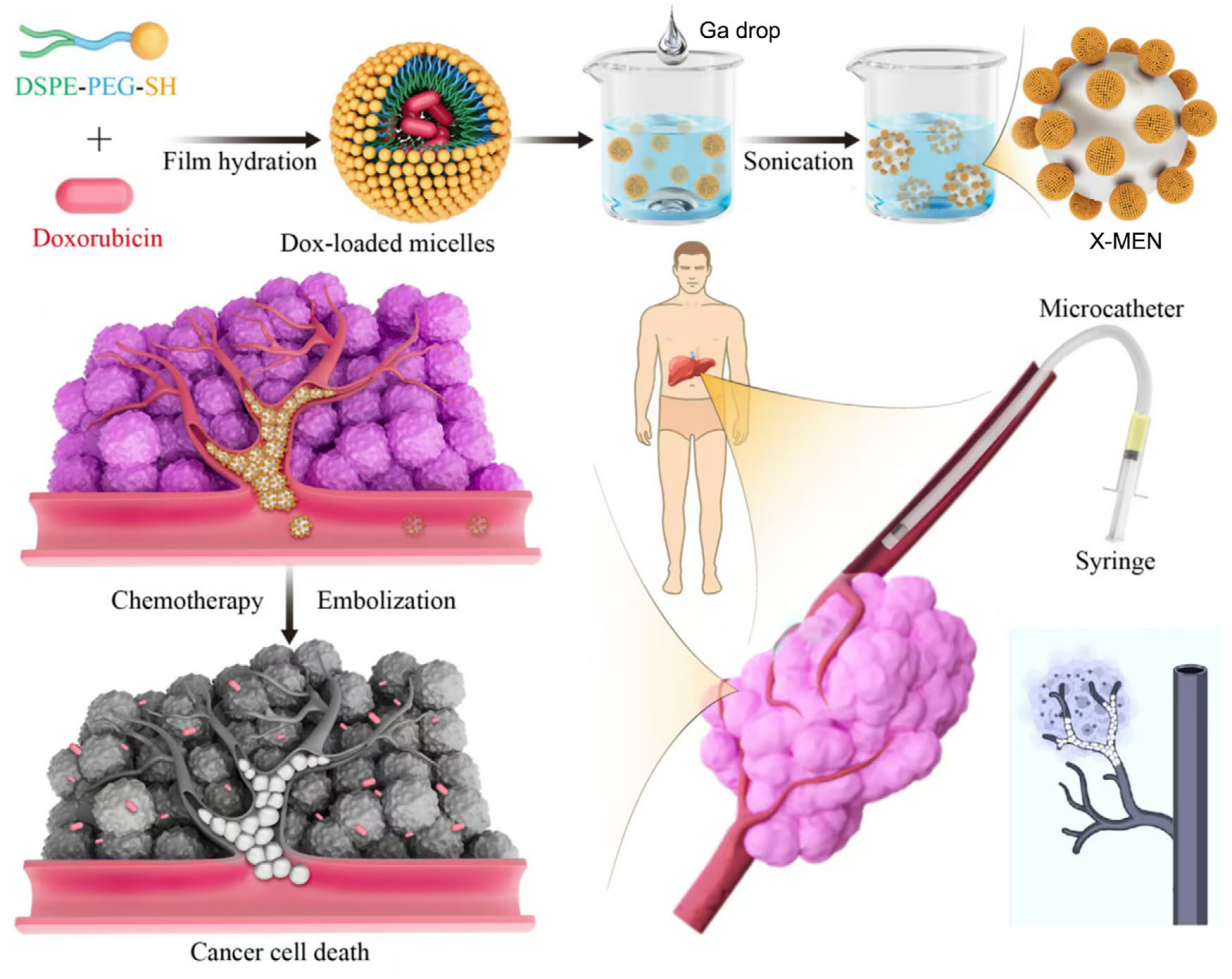
Schematic illustration of the fabrication and application of x‐ray visible liquid metal flexible embolization microspheres (X‐MEN) for image‐guided arterial embolization therapy.

Here, Gallium microspheres (GaMs) were generated by disrupting the surface tension of liquid metal Gallium using an ultrasound probe. Subsequently, novel x‐ray‐visible liquid metal flexible embolization microspheres (X‐MEN) were developed by modifying the surface of GaMs with doxorubicin‐loaded micelle (Dox‐m) for transcatheter arterial chemoembolization (TACE). Unlike conventional rigid solid microspheres, X‐MEN maintain a liquid core at physiological temperature (37°C). Their unique “external solid‐internal liquid” structure allows tight interparticle packing, as confirmed by scanning electron microscopy, resulting in efficient occlusion of tumor‐feeding arteries. X‐MEN demonstrated superior embolization performance compared to clinically used solid microspheres (e.g., Embosphere). Moreover, due to the radiopacity of gallium, X‐MEN enables real‐time visualization and localization at the tumor site using x‐ray or CT imaging. In the VX2 rabbit tumor model, X‐MEN exhibited significant therapeutic efficacy without notable systemic toxicity (Scheme 1). Moreover, drug‐free GaMs were used to treat benign prostatic hyperplasia in dogs, demonstrating long‐term embolization efficacy and biosafety of novel microspheres. Therefore, X‐MEN are promising visual persistent embolic agents with great potential in transcatheter arterial embolization treatment of tumors.

## 2 Results and Discussion

### 2.1 Preparation and Characterization of X‐MEN

Liquid metallic gallium can transform into gallium particles in a surfactant solution through ultrasonification. During the formation of gallium particles, thiolated‐ligands compete with the oxidation process, and the thiolated‐ligands are more likely to assemble onto the surface of the gallium. Therefore, thiolated 1,2‐Distearoyl‐sn‐glycero‐3‐phosphorylethanolamine‐Polyethylene glycol (DSPE‐PEG_2000_‐SH) was selected for the preparation of doxorubicin micelles (Dox‐m), enabling the surface of the Dox‐m to be covered with sulfhydryl groups (─SH). Through ultrasonic probing, the Dox‐m are assembled onto the surface of the gallium particles. The gallium particles with diameters in the range of 40–70 µm, namely X‐MEN, are obtained through filtration.

**FIGURE 1:**
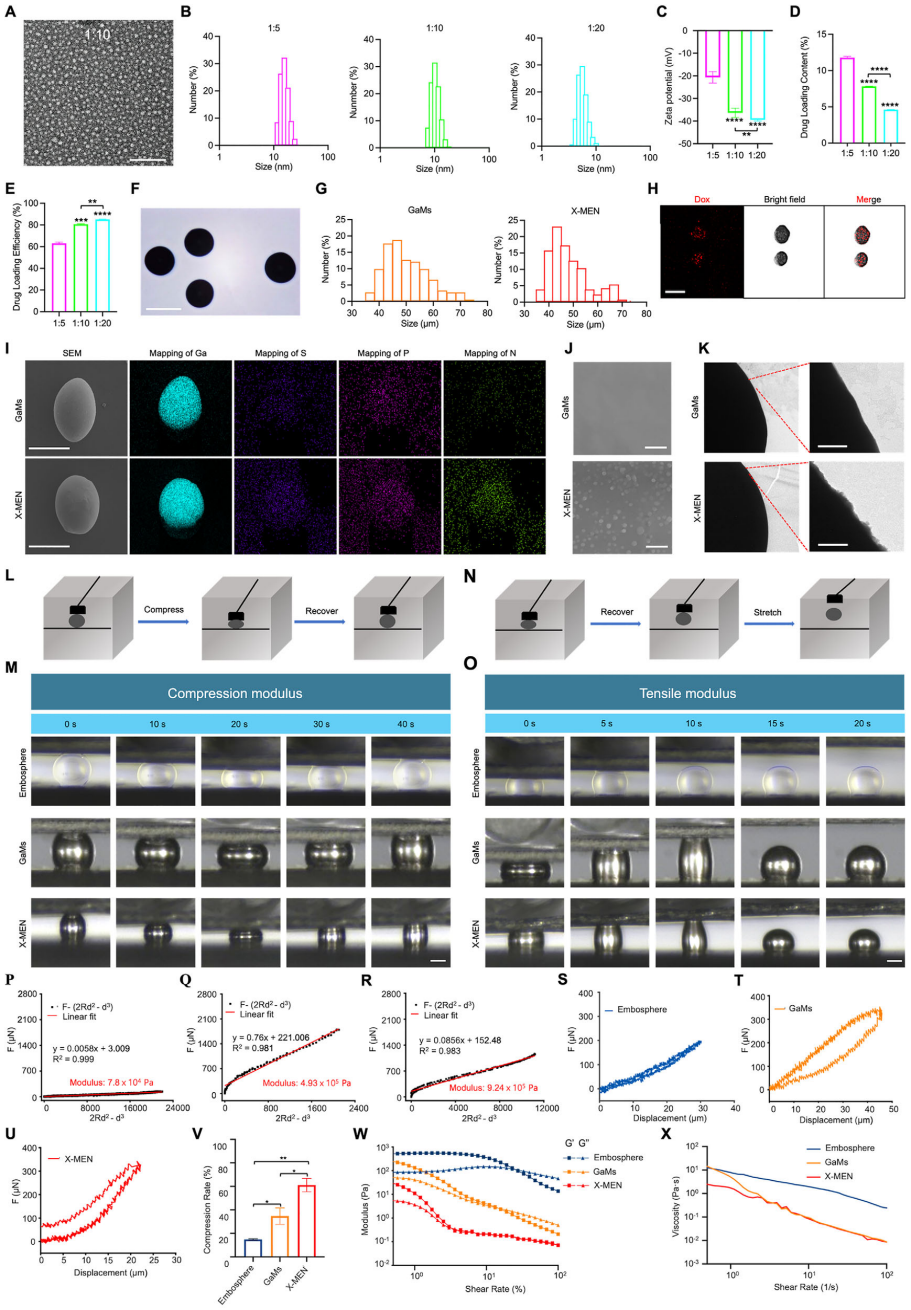
Preparation and characterization of X‐MEN. (A) TEM image of Dox‐m (1:10). Scale bar: 200 nm. (B) Size distributions of Dox‐m (1:5, 1:10, and 1:20). (C) Zeta potentials of Dox‐m (1:5, 1:10, and 1:20). Significant differences were evaluated by a two‐tailed unpaired *t‐*test. Data are presented as means ± SEM, *n* = 3. (D) Drug loading content of Dox‐m (1:5, 1:10, and 1:20). Significant differences were evaluated by a two‐tailed unpaired *t‐*test. Data are presented as means ± SEM, *n* = 3. ^****^ *p* < 0.001. (E) Drug loading efficiency of Dox‐m (1:5, 1:10, and 1:20). Significant differences were evaluated by a two‐tailed unpaired *t‐*test. Data are presented as means ± SEM, *n* = 3. ^**^ *p* < 0.01, ^***^ *p* < 0.005. (F) Optical microscope image of X‐MEN. Scale bar: 100 µm. (G) Size distributions of GaMs and X‐MEN. (H) Confocal microscope images of X‐MEN. Scale bar: 100 µm. (I) SEM and EDS images of GaMs and X‐MEN. Scale bars: 50 µm. (J) SEM images of GaMs and X‐MEN. Scale bars: 200 nm. (K) TEM images of GaMs and X‐MEN. Scale bars: 200 nm. (L) Schematic diagram of the compression process. (M) Images of the Embosphere, GaMs, and X‐MEN at different time points (0, 10, 20, 30, 40 s) during the compression process. Scale bar: 45 µm. (N) Schematic diagram of the tensile process. (O) Images of the Embosphere, GaMs, and X‐MEN at different time points (0, 5, 10, 15, 20 s) during the tensile process, Scale bar: 45 µm. (P) Compression modulus of the Embosphere calculated under the spherical model. (Q) Compression modulus of the GaMs calculated under the spherical model. (R) Compression modulus of the X‐MEN calculated under the spherical model. (S) Load‐displacement curve of the Embosphere. (T) Load‐displacement curve of the GaMs. (U) Load‐displacement curve of the X‐MEN. (V) Maximum compression ratio of Embosphere, GaMs, and X‐MEN. Significant differences were evaluated by a two‐tailed unpaired *t‐*test. Data are presented as means ± SEM, *n* = 3. ^*^ *p* < 0.05, ^**^ *p* < 0.001. (W) The modulus and shear strain curve of the Embosphere, GaMs, and X‐MEN. G’ = Storage Modulus, G″ = Loss Modulus. (X) Viscosity‐shear rate curve of the Embosphere, GaMs, and X‐MEN.

Doxorubicin micelles (Dox‐m) were prepared via the thin‐film hydration method at a Dox‐to‐DSPE‐PEG2000‐SH mass ratio of 1:10. The size and shape of Dox‐m were observed using transmission electron microscopy (TEM), as shown in Figure 1A. Similarly, Dox‐m with Dox and DSPE‐PEG_2000_‐SH mass ratios of 1:5 and 1:20 were also prepared, as depicted in Figure S1. The particle size (Figure 1B) and Zeta potential (Figure 1C) of all different formulations of micelles were measured using Malvern Zetasizer. As the proportion decreases, the particle size of Dox‐m decreases, and the negative potential increases. The drug loading content (DLC) and drug loading efficiency (DLE) of Dox‐m were analyzed, as illustrated in Figure 1D,E. Ultimately, it was determined that the optimal mass ratio of Dox to DSPE‐PEG_2000_‐SH during micelle preparation was 1:10. This formulation of Dox‐m exhibits a high DLC and a higher utilization rate of Dox. X‐MEN, observed as spherical in shape under an optical microscope (Figure 1F), were successfully prepared by treating Ga in Dox‐m through an ultrasonic probe. And 1 mL of X‐MEN can load 3.25 mg of Dox‐m.

The particle sizes of GaMs and X‐MEN are shown in Figure 1G, and the results are obtained from the percentage of their particle sizes within the optical microscopy images (Figure S2). The particle size of GaMs was 50.15 ± 8.07 µm, and the particle size of X‐MEN was 47.6 ± 7.32 µm. The excitation wavelength of Dox fluorescence is about 488 nm, and its emission wavelength is concentrated at 620 nm. Therefore, under these conditions, it is possible to determine whether X‐MEN are drug‐loaded by observing whether they have fluorescence using a confocal laser scanning microscope. The results (Figure 1H) showed that X‐MEN were visible in the bright field, and red fluorescence was observed when switching to the dark field (fluorescence channel). In the merged image, the position of X‐MEN perfectly matched the red fluorescence, confirming the presence of Dox‐m on the X‐MEN surface. Under the same conditions, no fluorescence was observed in GaMs (Figure S3). Scanning electron microscopy (SEM) observations of Embosphere, GaMs, and X‐MEN (Figure S4) indicated that the particle sizes of these microspheres were consistent with those observed under optical microscopy. Interestingly, X‐MEN were able to adhere more closely together, which is notably different from the solid microspheres (Embosphere). The morphology of these microspheres was no longer spherical but exhibited a soft “external solid‐internal liquid” structure.

The results of the elemental composition analysis of GaMs and X‐MEN using energy dispersive spectroscopy (EDS) are shown in Figure 1I. The EDS map of X‐MEN reveals the presence of sulfur, phosphorus, and nitrogen elements. Sulfur and phosphorus are elements present in the DSPE‐PEG_2000_‐SH used in the preparation of Dox‐m, and their presence proves that Dox‐m was successfully loaded onto X‐MEN. Nitrogen, a common element shared by DSPE‐PEG_2000_‐SH and Dox, was not observed in the EDS map of GaMs, ruling out the possibility of other substances interfering with the above results. Nanoparticles were found on the surface of the X‐MEN, as shown in Figure 1J, and these were similar in size to the Dox‐m previously observed by TEM, while none were observed on GaMs. These findings indicate that Dox‐m is present on the surface of X‐MEN. In addition, GaMs and X‐MEN were observed by TEM (Figure 1K). GaMs have smooth and regular edges because of their superficial oxide layer. In contrast, the rough edges of X‐MEN are due to the involvement of Dox‐m, which carries sulfhydryl groups, in the composition of its surface layer.

The compression and tensile properties of Embosphere, GaMs, and X‐MEN were systematically characterized using Microtester G2. Figure 1L illustrates the entire compression process of the sample in the Microtester G2, from uncompressed to compressed, and finally recovered. The compression state change plots show that compared to Embosphere, both GaMs and X‐MEN exhibit comparable compression properties, as well as a structure that can recover quickly, suggesting some flexible properties (Figure 1M). Figure 1N showed the entire stretching process of the sample from compression, recovery, to stretching in the Microtester G2. GaMs and X‐MEN were able to stretch easily under the traction of the sample rod, demonstrating excellent viscosity, whereas the commercial microspheres (Embosphere) failed to achieve effective stretching (Figure 1O). This may be related to the tension of the material itself. For example, when liquid (like water) is placed between two glass plates, it can be adsorbed at first, but if fixed, it cannot exhibit the same effect. This suggests that GaMs and X‐MEN possess liquid properties.

**FIGURE 2:**
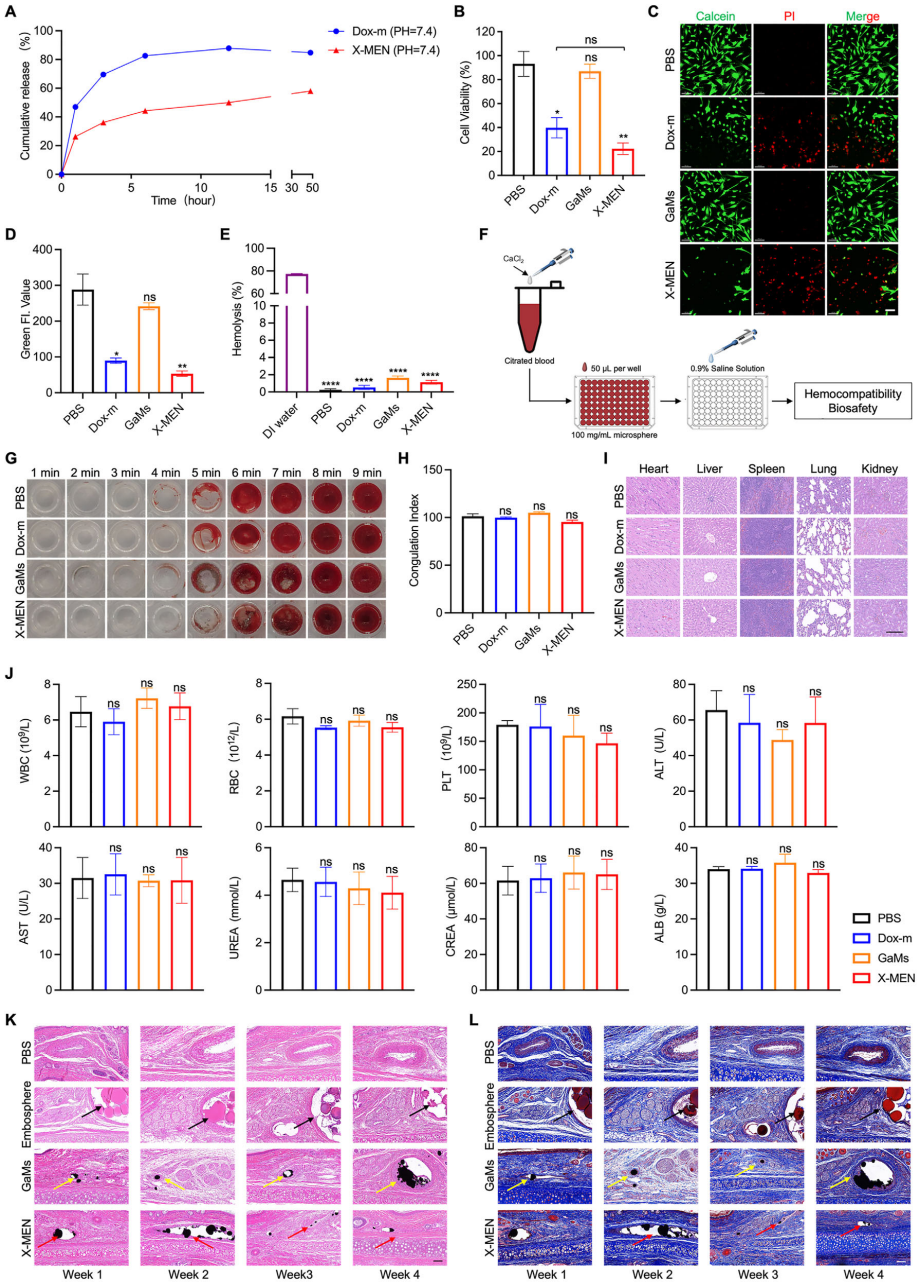
In vitro and in vivo evaluation of X‐MEN. (A) Cumulative Dox release from Dox‐m and X‐MEN in PBS at pH 7.4. (B) Cell viability of VX2 cells after exposure to Dox‐m, GaMs, and X‐MEN for 24 h. Significant differences were evaluated by a two‐tailed unpaired *t‐*test. Data are presented as means ± SEM, *n* = 3, ns *p* > 0.05, ^*^ *p* < 0.05, ^**^ *p* < 0.001. (C) Calcein/PI staining of VX2 cells co‐culture with PBS, Dox‐m, GaMs, and X‐MEN for 24 h. (D) Green FI. Value of VX2 cells after Calcein/PI staining. Significant differences were evaluated by a two‐tailed unpaired *t‐*test. Data are presented as means ± SEM, *n* = 3, ns *p* > 0.05, ^*^ *p* < 0.05, ^**^ *p* < 0.001. (E) Hemolysis test of Dox‐m, GaMs, X‐MEN. Significant differences were evaluated by a two‐tailed unpaired *t‐*test. Data are presented as means ± SEM, *n* = 3, ^****^ *p* < 0.001. (F) Schematic diagram of the coagulation test. (G) Blood coagulation test of Dox‐m, GaMs, X‐MEN. (H) Blood coagulation index of Dox‐m, GaMs, X‐MEN. Significant differences were evaluated by a two‐tailed unpaired *t‐*test. Data are presented as means ± SEM, *n* = 3, ns *p* > 0.05. (I) Histological photomicrographs of primary organs after treatment with PBS, Dox‐m, GaMs, and X‐MEN for 7 days, scale bar: 100 µm. (J) The blood routine examination, liver and kidney function examination, and blood coagulation examination after treatment with PBS, Dox‐m, GaMs, and X‐MEN for 7 days. Significant differences were evaluated by a two‐tailed unpaired *t‐*test. Data are presented as means ± SEM, ns *p* > 0.05. (K) Hematoxylin and eosin (H&E) staining of rabbit ear tissues at 1, 2, 3, and 4 weeks after embolization. The black arrows indicate Embosphere, the yellow arrows indicate GaMs, and the red arrows indicate X‐MEN. Bar:100 µm. (L) Masson's trichrome staining of rabbit ear tissues at 1, 2, 3, and 4 weeks after embolization (Fig K and L are slices taken from the same location of the same tissue and stained differently). The black arrows indicate Embosphere, the yellow arrows indicate GaMs, and the red arrows indicate X‐MEN. Bar:100 µm.

Furthermore, quantitative analysis of the compressive modulus using spherical models demonstrated that GaMs and X‐MEN possess significantly higher compressive moduli compared to Embosphere. This result highlighted that GaMs and X‐MEN have enhanced stability, superior resistance to compression, and retained structural integrity under high compressive loads (Figure 1P–R). As illustrated in Figure 1S–U, the load‐displacement curves of GaMs and X‐MEN exhibited distinct hysteresis loops during the compression‐recovery cycle, confirming their intrinsic viscoelasticity. In contrast, Embosphere displayed no detectable hysteresis loop, indicating poor viscoelastic behavior, which aligns with the observations in Figure 2O. Under identical external force conditions, maximum compression ratio tests were conducted for Embosphere, GaMs, and X‐MEN (Figure S5). Benefiting from their unique core‐liquid and shell‐solid structure, GaMs and X‐MEN exhibited significantly higher compression ratios compared to Embosphere, as shown in Figure 1V. As depicted in Figure 1W, Embosphere exhibited dominant elasticity, behaving as a flexible material across both low and high‐frequency ranges, with weak viscosity; GaMs also exhibited dominant elasticity, with more pronounced viscoelastic properties; X‐MEN showed dominant viscosity, with slight elasticity at low frequencies, but as the frequency increases, the material's viscous behavior becomes dominant, resembling a liquid or gel‐like substance. The viscosity of Embosphere, GaMs, and X‐MEN significantly decreased with an increase in shear rate, which was beneficial for their delivery through catheters (Figure 1X).

### 2.2 In Vitro and In Vivo Evaluation of X‐MEN

To investigate drug release behavior, the dialysis method was employed to study the release of Dox from Dox‐m and X‐MEN in a medium with pH 7.4 at 37°C over 48 h, as shown in Figure 2A. PBS solution with a pH of 7.4 was prepared as the release medium to simulate normal physiological conditions. In PBS at pH 7.4, both Dox‐m and X‐MEN exhibited favorable sustained‐release characteristics of Dox, which is crucial for TACE therapy, as long‐term retention and continuous drug release at the embolization site are key factors for achieving effective localized chemotherapy. In addition, the release behavior of X‐MEN over 7 days was further investigated (Figure S6), and the cumulative release of Dox reached approximately 60.52 ± 0.20% in the medium at pH 7.4.

Given that X‐MEN are designed for intravascular embolization, assessing their biocompatibility is paramount. The cell viability test by the CCK‐8 assay was performed usingVX2 cells. As shown in Figure 2B, after 24 h of co‐culture, the cell viability of the Dox‐m and X‐MEN groups decreased to 39.77 ± 8.46% (*p* = 0.0164) and 22.34 ± 4.81% (*p* = 0.0035), respectively. Although the X‐MEN group exhibited a lower cell viability than the Dox‐m group, the difference between the two groups was not statistically significant (*p* = 0.1479). In contrast, the cell viability of the GaMs group was 86.97 ± 5.95%, which was comparable to that of the PBS group (93.17 ± 10.4%) with no statistically significant difference (*p* = 0.0628). Compared to other studies [42], the cell viability co‐cultured with GaMs has decreased. This may be due to the physical toxicity caused by the larger particle diameter in this study. As illustrated in Figure 2C,D, live/dead staining revealed minimal cell death in the PBS and GaMs groups, while extensive cell death was evident in the Dox‐m and X‐MEN groups. As depicted in Figure S7, the results are consistent when the dose is reduced by half. Therefore, GaMs have low cytotoxicity, while the cytotoxicity of X‐MEN originates from the Dox they release. Meanwhile, to confirm the blood compatibility of X‐MEN, we conducted hemolysis and coagulation tests in vitro. As shown in Figure 2E, the hemolysis rates of GaMs and X‐MEN were 1.7% and 1.1%, respectively. Then we evaluated the blood compatibility of the material by measuring the clotting time of rabbit whole blood in contact with Dox‐m, GaMs, and X‐MEN (Figure 2F). As shown in Figure 2G, the clotting initiation time remained consistent at approximately 4 min across all groups, indicating no significant alteration in coagulation kinetics compared to PBS. As shown in Figure 2G, the clotting initiation time remained consistent at approximately 4 min across all groups, indicating no significant alteration in coagulation kinetics compared to PBS. Meanwhile, the safety of X‐MEN was validated in rabbits. Pathologic examinations of the major organs of rabbits 7 days after embolization were performed. As shown in Figure 2G, the clotting initiation time remained consistent at approximately 4 min across all groups, indicating no significant alteration in coagulation kinetics compared to PBS. This suggests that X‐MEN, as an intravascular embolic agent, did not embolize normal organs through the capillary system. Hematological parameters (WBC, RBC, PLT) and serum biochemical markers for renal function (UREA, CREA, ALB) and liver function (ALT, AST) were analyzed. As shown in Figure 2J, no significant changes were observed in any of the measured parameters post‐treatment compared to the PBS group, indicating the absence of systemic toxicity Histological analysis using H&E and Masson's trichrome staining revealed time‐dependent peri‐vascular tissue responses following embolization, as shown in Figure 2K,L. Compared with the control group, Embosphere induced only mild and transient inflammatory infiltration with minimal collagen deposition, resulting in the formation of a thin and stable fibrous capsule by week 4. GaMs elicited peri‐vascular tissue responses comparable to those of Embosphere, showing similarly mild inflammation and limited collagen accumulation. GaMs and X‐MEN elicited peri‐vascular tissue responses comparable to those of Embosphere, exhibiting similarly mild inflammation and limited collagen accumulation, without evidence of progressive inflammation or tissue damage. These results indicate that the liquid metal microspheres exhibit overall favorable histocompatibility.

### 2.3 High‐Resolution Computed Tomography Imaging of X‐MEN

**FIGURE 3:**
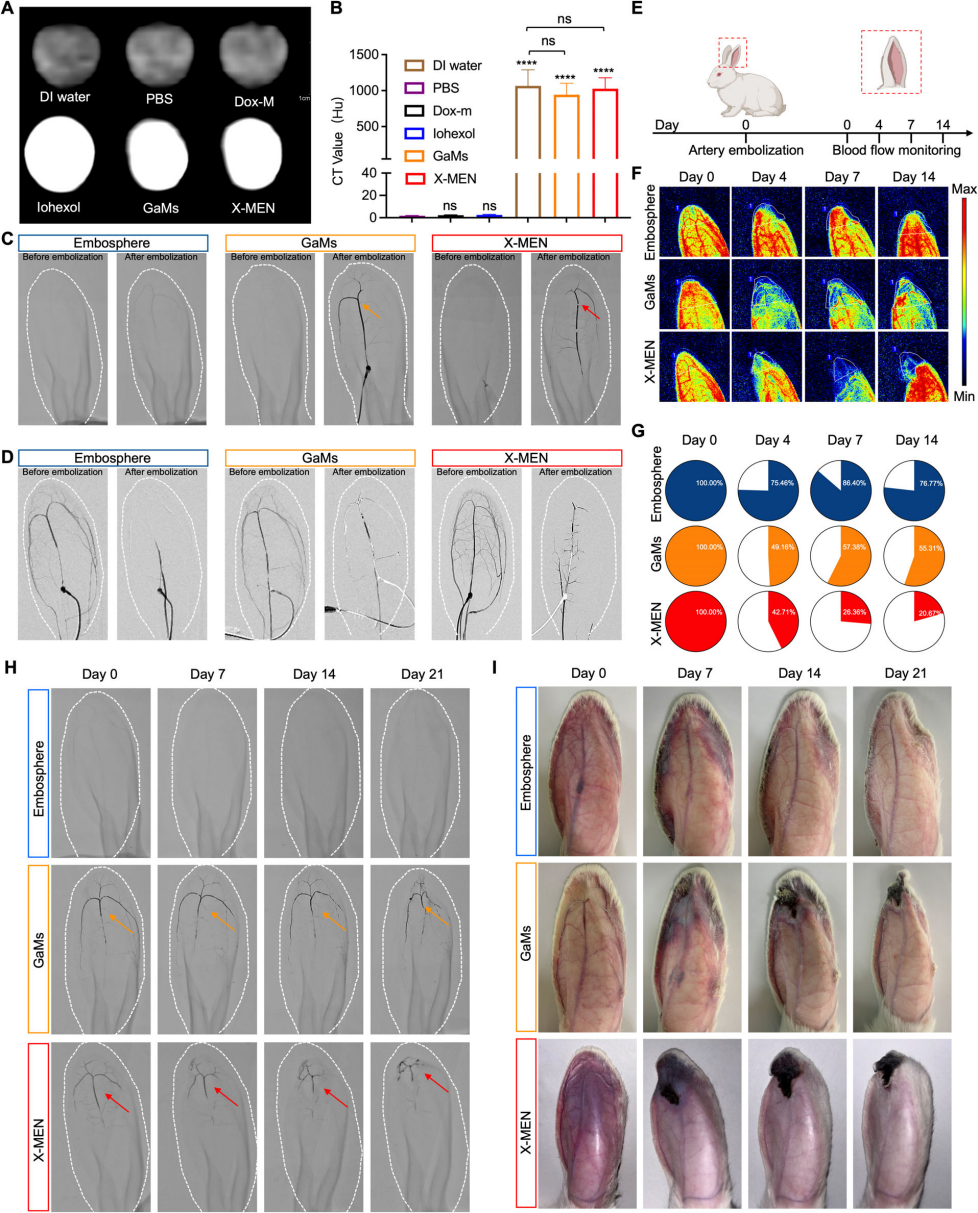
High‐resolution imaging and embolization effect of X‐MEN. (A) CT images of DI water, PBS, Dox‐m, Iohexol, GaMs, and X‐MEN. (B) CT values of DI water, PBS, Dox‐m, Iohexol, GaMs, and X‐MEN. Significant differences were evaluated by a two‐tailed unpaired *t‐*test. Data are presented as means ± SEM, *n* = 3, ns *p* > 0.05, ^****^ *p* < 0.001. (C) Single‐snapshot radiography of the ear before and after embolization. The arrowheads indicate the GaMs and X‐MEN inside the blood vessels. (D) Images from digital subtraction angiography (DSA) of the ear before and after embolization. After embolization, the vascular contrast enhancement is significantly reduced. (E) Schematic diagram illustrating the monitoring of rabbit ear blood flow before and after embolization. (F) Blood perfusion images of rabbit ears before and after embolization. Red represents rich blood flow, while blue represents low blood flow. (G) Changes in blood perfusion of rabbit ears before and after embolization. (H) Single‐snapshot radiography of rabbit ears at different time points after embolization. GaMs and X‐MEN (arrowheads) can be continuously observed over 21 days, whereas Embosphere cannot. (I) Photographs of rabbit ears at different time points after embolization.

The inherent radiopacity of microspheres facilitates real‐time monitoring by interventional radiologists during the embolization procedure. Fabricated from high‐density liquid metal Ga, X‐MEN can be visualized under x‐ray imaging without the need for additional contrast agents. To evaluate the radiopacity of X‐MEN, we performed CT scans and analyzed their CT values. As shown in Figure 3A, Iohexol, GaMs, and X‐MEN appeared as high‐density regions, whereas deionized water, PBS, and Dox‐M exhibited low attenuation. Quantitative analysis of CT values (HU) is presented in Figure 3B. The CT value of GaMs was 943.7 ± 158.4, and the CT value of X‐MEN was 1026.3 ± 150.6. Compared with the CT value of Iohexol (1067.4 ± 223.3), there was no significant statistical difference (*p* = 0.6767, *p* = 0.6253). CT values correlate with x‐ray attenuation, serving as a metric for radiopacity. These results indicate that liquid metal‐derived microspheres possess radiopacity comparable to clinical contrast agents like Iohexol. To further confirm the in vivo radiopacity of X‐MEN, we performed embolization of the central artery of the rabbit ear and a single‐snapshot image of the rabbit ear before and after embolization. As illustrated in Figure 3C, in the single‐snapshot image after embolization, the positions of GaMs and X‐MEN could be observed, whereas Embosphere, a clinically used embolic microsphere, could not be detected. As illustrated in Figure 3D, the images before embolization fully displayed the arteriovenous system of the rabbit ear. After embolization, the images from DSA of Embosphere, GaMs, and X‐MEN only showed partial arteries, indicating that they had successfully blocked the rabbit ear artery. Laser speckle perfusion imaging (PeriCam PSI) monitored blood flow changes (Figure 3E). Notably, the X‐MEN group exhibited significantly lower blood flow recovery compared to the Embosphere and GaMs groups (Figure 3F,G). Meanwhile, the single‐snapshot images of the rabbit ears were performed to observe changes in the distribution and position of these embolic agents in vivo. As shown in Figure 3H, GaMs and X‐MEN remained in their original position without movement for 21 days after embolization. However, since Embosphere lacked radiopacity, its position could not be observed in the single‐snapshot images. The changes in the rabbit ears after embolization were continuously observed and recorded for 21 days (Figure 3I). Rabbit ear necrosis was more severe after embolization of X‐MEN and GaMs compared to Embosphere. This indicates that GaMs and X‐MEN can serve as a stable vascular embolic agent.

### 2.4 Embolization Efficacy of X‐MEN in VX2 Rabbit Model

**FIGURE 4:**
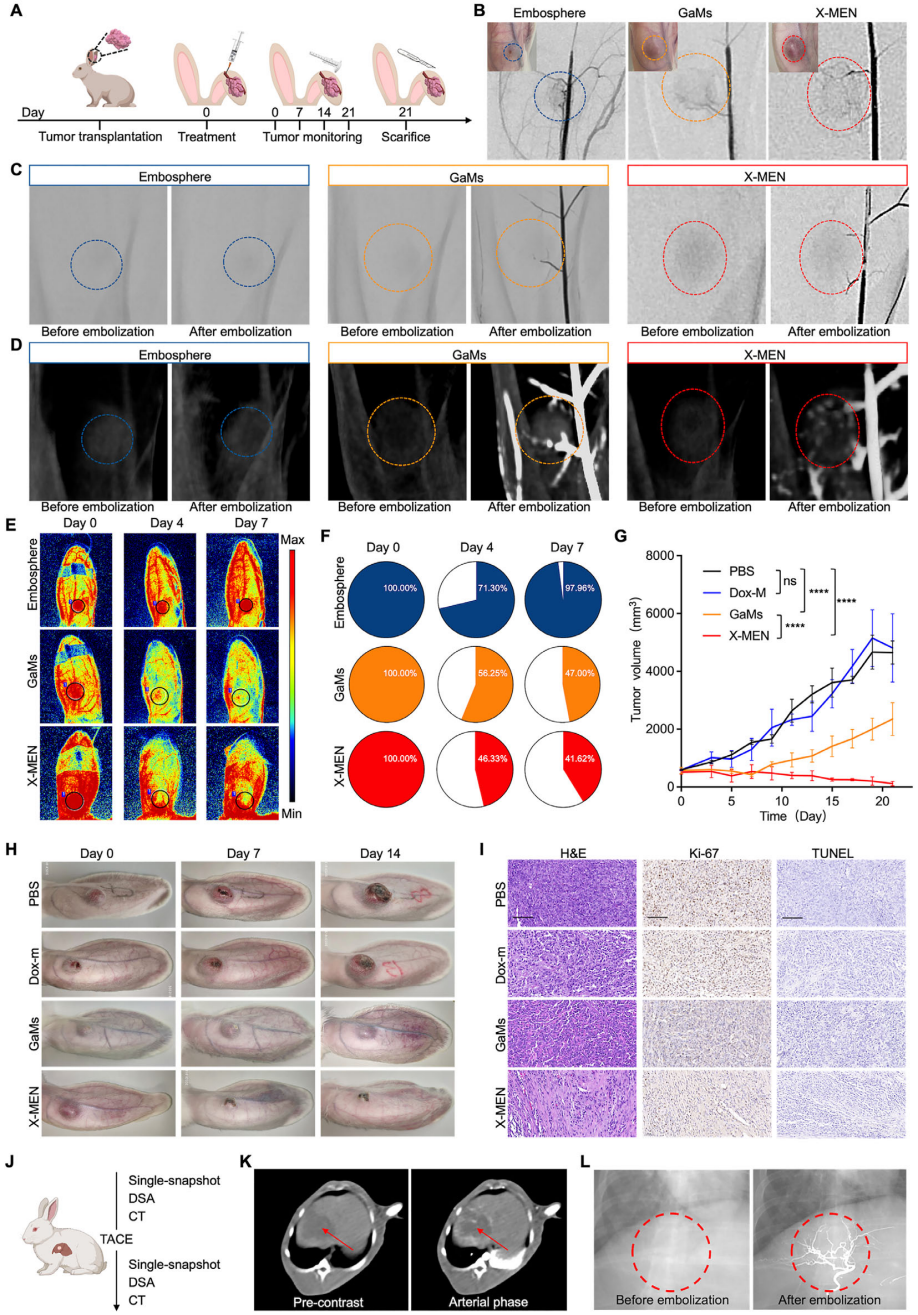
Embolization Efficacy of X‐MEN in VX2‐xenograft rabbit model. (A) Schematic diagram of tumor treatment by TACE with X‐MEN. (B) Photograph of the rabbit VX2‐tumor, and DSA images of the rabbit auricular artery. The area within the circle indicates the location of the tumor. (C) Single‐snapshot images before and after embolization with Embosphere, GaMs, and X‐MEN. (D) CT images before and after embolization with Embosphere, GaMs, and X‐MEN. (E) Blood perfusion images of rabbit ears before and after embolization. Red represents high blood flow, while blue represents low blood flow. (F) Changes in blood perfusion of rabbit ears before and after embolization. (G) Tumor growth curves of rabbits. Significant differences were evaluated by a two‐tailed unpaired *t‐*test. Data are presented as means ± SEM, *n* = 3, ns *p* > 0.05, ^****^ *p* < 0.0001. (H) The photographs of rabbit ears before and after tumor treatment. (I) Histological photomicrographs of H&E, Ki‐67, and TUNEL‐stained tumor tissue, scale bars: 100 µm. (J) Therapeutic workflow for the rabbit liver tumor model. (K) Contrast‐enhanced CT images of rabbit liver cancer, with arrows indicating the tumor. (L) Images from single‐snapshot radiography of the tumor before and after embolization. The circular markers indicate X‐MEN distributed within the blood vessels.

As shown in Figure 4A, the effectiveness of X‐MEN as an embolic agent for TACE was validated in the rabbit ear tumor model. By implanting a tumor tissue suspension into the subcutaneous area around the central artery of the rabbit ear, a rabbit VX2 tumor model was established (Figure S8). Three weeks after transplantation, the tumor volume reached approximately 10 × 10 × 10 mm^3^. Before treatment, there was no difference in tumor volume among the four groups. As depicted in Figure 4B, a DSA examination was performed on the rabbit ears in each group before embolization, where tumor staining and the main feeding artery were observed. Compared with pre‐embolization, the post‐embolization single‐snapshot images (Figure 4C) clearly showed the presence of GaMs and X‐MEN at the location of the tumor's main feeding artery, while Embosphere was not visible. The post‐embolization CT images (Figure 4D) showed a significant increase in radiopacity in the tumor region for the GaMs and X‐MEN groups. The 3D reconstructed CT images (Figure S9) also demonstrated the presence of microspheres inside the tumor. Using the PeriCam PSI system to monitor blood flow changes in the tumor region after embolization, it was found that the blood flow recovery in the X‐MEN and GaMs groups was significantly lower than that in the Embosphere group, as illustrated in Figure 4E,F. This was consistent with the observation in the rabbit ear without a tumor. Changes in rabbit ear tumor volume were recorded for each group over 21 days (Figure S10). As shown in Figure 4G, the tumor growth rate in the Dox‐m group was almost the same as that in the PBS group. On day 7, the tumor volume increased from 586.4 ± 23.57 to 1301.7 ± 385.4 mm^3^ and from 594.5 ± 22.76 to 1543.5 ± 71.56 mm^3^ in the two groups, respectively, and there was no significant difference in the change in tumor volume between the two groups (*p* = 0.8115). In contrast, the tumor volumes in the GaMs and X‐MEN groups in the first 7 days decreased from 570.1 ± 107.6 to 423.3 ± 50.60 mm^3^ and increased from 512.6 ± 69.01 to 532.4 ± 242.5 mm^3^, respectively. The tumor growth rate was inhibited in both the X‐MEN and GaMs groups compared to the PBS group (*p* = 0.0002, *p* = 0.0162). Over the next 7 days, the tumor volume in the GaMs group rapidly increased to 1075.6 ± 216.6 mm^3^, whereas the tumor volume in the X‐MEN group decreased to 387.0 ± 172.8 mm^3^, with the X‐MEN group showing a better therapeutic effect. By the end of the observation (day 21), the tumor volume in the X‐MEN group continued to decrease to 118.7 ± 76.36 mm^3^. Representative photographs of tumor changes in each group are shown in Figure 4H. As illustrated in Figure 4I, histopathological analyses, including H&E, Ki‐67, and TUNEL staining, were performed on the tumor tissues after 7 days of treatment to evaluate the therapeutic effects on the tumors. H&E staining results showed that the number of tumor cells in the X‐MEN group was significantly lower than in the other three groups. Ki‐67 staining results indicated that the proportion of Ki‐67‐positive cells in the X‐MEN group was significantly lower than in the PBS and Dox‐m groups, with the X‐MEN group showing the lowest proportion. TUNEL staining results showed that the proportion of TUNEL‐positive cells in the X‐MEN and GaMs groups was significantly higher than in the PBS and Dox‐m groups, with the X‐MEN group showing the highest proportion. This indicated that the therapeutic effects of the GaMs group and X‐MEN group were significantly better than those of the PBS group and Dox‐m group, among which the X‐MEN group had the best therapeutic effect.

In addition, a rabbit liver cancer model was established to verify the embolic performance of X‐MEN, as shown in Figure 4J. Tumor implantation was confirmed by contrast‐enhanced CT (Figure 4K). Compared with the pre‐embolization status, the post‐embolization DSA images showed that the tumor staining completely disappeared, as depicted in Figure S11. This indicates that X‐MEN successfully embolized the tumor‐feeding arteries. Single‐snapshot images (Figure 4L) before and after embolization showed that X‐MEN filled various nutrient‐supplying arteries, achieving stepwise embolization. In the CT images (Figures S12 and S13), the distribution of X‐MEN could also be observed. These results demonstrate the great potential of X‐MEN as an embolic agent for TACE in the treatment of liver cancer.

### 2.5 The Long‐Term Embolization Effect and Biosafety in the Dog Model

**FIGURE 5:**
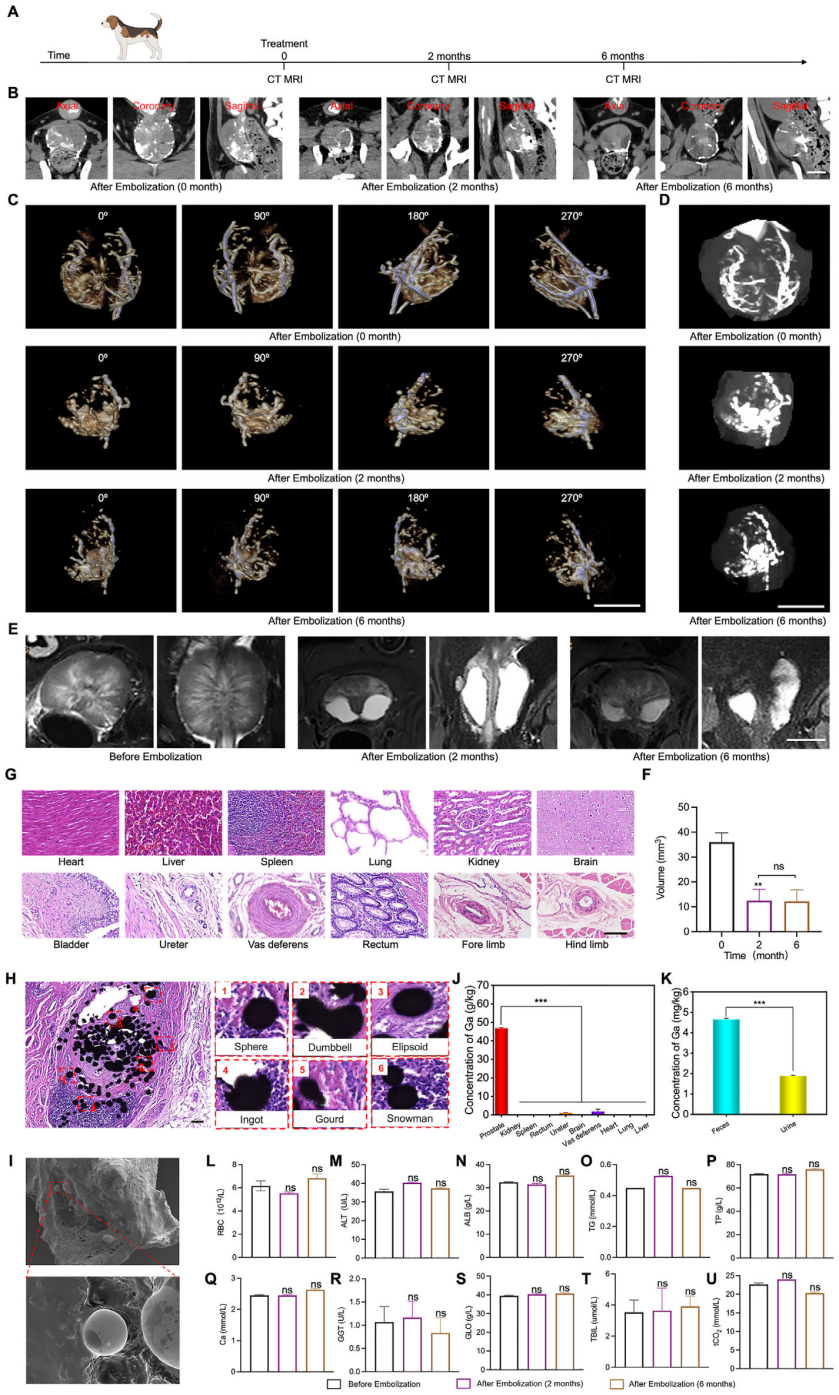
The long‐term embolization effect and biosafety in the Beagle dog model. (A) Flowchart of Beagle dog experiments. (B) CT images after 2 and 6 months post‐embolization in the prostatic hyperplasia model. Scale bar: 2 cm. (C) 3D CT images after 2 and 6 months post‐embolization in the prostatic hyperplasia model. Scale bar: 3 cm. (D) MIP CT images after 2 and 6 months post‐embolization in the prostatic hyperplasia model. Scale bar: 3 cm. (E) MRI images before embolization and 2 and 6 months post‐embolization in the prostatic hyperplasia model. Scale bar: 2 cm. (F) Prostate volume change histogram. Significant differences were evaluated by a two‐tailed unpaired *t‐*test. Data are presented as means ± SEM, *n* = 3, ns *p* > 0.05, ^**^ *p* < 0.001. (G) Histological photomicrographs of primary organs after embolization 6 months. Scale bar: 100 µm. (H) Histological photomicrographs of the prostate after embolization 6 months. Scale bar: 100 µm. (I) SEM images of tissue samples 6 months after canine prostate vascular embolization. Scale: 50 µm. (J) Ga content in different organs. (K) Ga content in feces and urine. (L–U) Blood tests in the prostatic hyperplasia model with and without GaMs treatment. Significant differences were evaluated by a two‐tailed unpaired *t‐*test. Data are presented as means ± SEM, *n* = 3, ns *p* > 0.05; ^**^ *p <* 0.01.

We evaluated the long‐term stability of GaMs as embolic agents in beagle dogs (Figure 5A). Results demonstrated that these microspheres remained at the embolization site for at least six months, indicating sustained embolic efficacy. Due to the radiopacity of these microspheres, their distribution in the prostate could be observed by CT, as shown in Figure 5B. These microspheres were localized in the blood vessels around and inside the prostate. A comparison between 3D‐CT and maximum intensity projection images taken at 2 and 6 months post‐embolization revealed that these particles remained stable in the embolized area (Figure 5C,D). Figure 5E shows that the pre‐embolization MRI indicated significantly enlarged canine prostate, however, at 2 and 6 months post‐embolization, the MRI images revealed obvious liquefactive necrosis in both lobes of the prostate. 2 months after embolization, the volume of the hypertrophic prostate decreased from 36.0 ± 3.7 to 12.4 ± 4.6 cm^3^, and it remained stable with a volume of 12.2 ± 4.5 cm^3^ after 6 months, as illustrated in Figure 5F. In Figure 5G, the heart, liver, spleen, lung, kidney, brain, bladder, ureter, vas deferens, rectum, forelimbs, and hindlimbs all appeared normal, with no microspheres observed. The histopathological section of the prostate (Figure 5H) shows that long‐term embolization resulted in sustained ischemia and foreign body reactions, leading to gradual damage or even disappearance of the vascular wall structure. Notably, the microspheres remained stably present and exhibited various morphologies, including spherical, dumbbell‐shaped, ellipsoidal, ingot‐shaped, gourd‐shaped, and snowman‐like forms. SEM images of tissue samples 6 months after embolization further confirmed that the spherical structure of the microspheres remained intact during the 6‐month implantation period, and there was no significant fragmentation or degradation (Figure 5I). This is consistent with previous SEM observations, fully demonstrating that the liquid metal‐prepared microspheres have good deformability and stability in vivo. Besides, measurements of gallium (Ga) content in various organs (Figure 5J) showed that the prostate contained significantly more gallium than other organs. This indicates that the vast majority of microspheres successfully entered the prostate and achieved effective embolization. The presence of gallium was detected in the collected feces and urine of dogs, with higher levels of Ga found in feces compared to urine (Figure 5K). This indicates that the majority of Ga in the body is excreted through feces, thereby reducing the burden on the kidneys. Additionally, the blood test results before and after embolization at 2 and 6 months (Figure 5L–U) showed that the liquid metal particles exhibited good biocompatibility in vivo. Therefore, the concept of designing a liquid metal microsphere integrating embolization, visualization, and drug release for TACE is well‐supported.

### 2.6 Immunocompatibility Evaluation of Ga Microspheres

**FIGURE 6:**
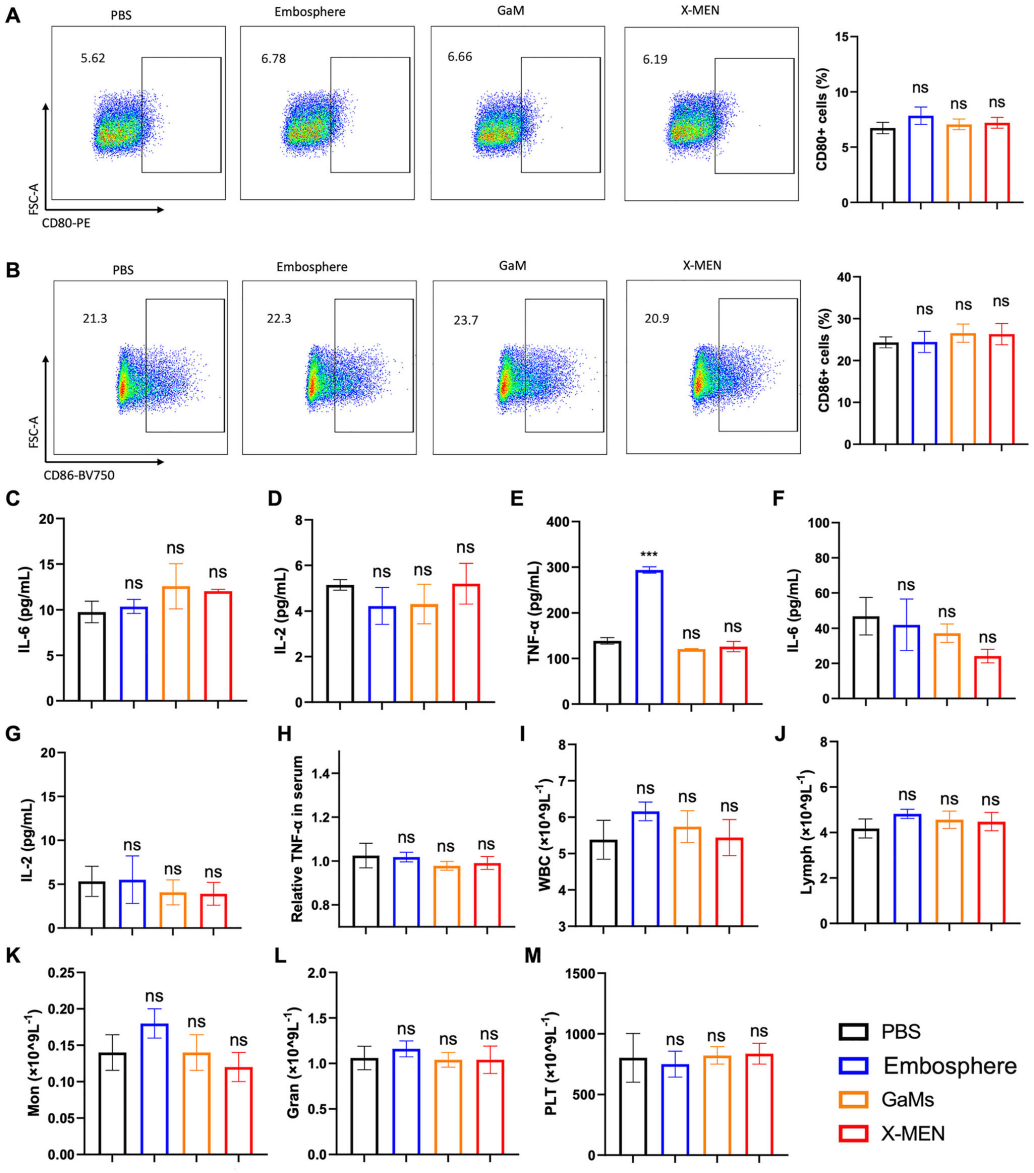
Immunocompatibility evaluation of Ga microspheres. (A, B) Representative flow cytometry plots and corresponding quantification of CD80^+^ and CD86^+^ in RAW264.7 cells. (C–E) Quantification of inflammatory cytokines in RAW264.7 cell culture supernatants incubated with X‐MEN, GaMs, Embosphere, and PBS. (F–H) Quantification of inflammatory cytokines in mouse blood serum 24 h after injecting X‐MEN, GaMs, Embosphere, and PBS. (I–M) Hematological analysis of C57BL/6J mice 24 h after injecting X‐MEN, GaMs, Embosphere, and PBS.

To further evaluate the immunocompatibility of X‐MENs and GaMs, we conducted both in vitro and in vivo assessments. For the in vitro study, we selected RAW264.7 cells, a macrophage cell line sensitive to inflammatory stimuli, to assess the immunogenicity of X‐MENs and GaMs. The gating strategy for flow cytometry is presented in Figure S14. First, we examined the polarization phenotype of RAW264.7 cells after a 24‐h incubation with X‐MENs and GaMs. The proportions of CD80^+^ and CD86^+^ cells in the X‐MEN and GaMs groups showed no significant differences compared to those in the Embosphere and PBS groups (Figure 6A,B). Subsequently, we collected the cell culture supernatants and found that the levels of key pro‐inflammatory cytokines (IL‐6, IL‐2, and TNF‐α) in the X‐MEN and GaMs groups were comparable to those in the PBS control group, with no statistically significant differences observed (Figure 6C–E). We then conducted in vivo experiments using C57BL/6J mice to further confirm the immunocompatibility of X‐MENs and GaMs. Blood samples were collected 24 h post‐administration. Consistent with our in vitro findings, the serum levels of major inflammatory cytokines (IL‐6, IL‐2, and TNF‐α) in the X‐MEN and GaMs groups remained comparable to those in the PBS control group, showing no statistically significant differences (Figure 6F–H). Furthermore, hematological analysis performed 24 h post‐administration provided additional evidence of the favorable immunocompatibility of X‐MENs and GaMs (Figure 6I–M).

## 3 Conclusion

The improvement of interventional therapy outcomes is inseparable from the development of embolic materials. Ideal embolic agents for tumor TACE generally need to possess characteristics such as biocompatibility, ease of catheter delivery, material stability, radiopacity, and drug delivery capability. Due to the high density of gallium, gallium‐based microspheres exhibit poor suspension properties in solution. Therefore, it was initially hypothesized that these microspheres might not pass through the microcatheter well. However, the microspheres successfully reached the target vessels and achieved complete embolization, proving our concerns unnecessary. Another challenge in our research was achieving drug loading and release from the microspheres. These microspheres are non‐hollow, so they cannot accommodate drugs internally, nor can they load drugs through ion exchange like DC Bead. Therefore, we encapsulated chemotherapy drugs within micelles, allowing the micelles to be grafted onto the surface of the microspheres via gallium–sulfur bonds. After embolization, the drug within the micelles is released through diffusion.

In summary, we have designed a liquid metal drug‐loaded microsphere with excellent biosafety. Unlike common solid microspheres, liquid metal microspheres maintain a stable liquid core/solid shell structure at 37°C. By functionalizing the surface of the microspheres, Dox‐m was successfully loaded onto the microspheres, and the drug could be slowly released. Owing to the radiation impermeability of the metal itself, the microspheres can be monitored in real‐time during arterial embolization therapy. As expected, we successfully embolized the rabbit auricular artery with X‐MEN under DSA. Furthermore, the effectiveness and safety of X‐MEN were validated in the treatment of rabbit liver cancer models. X‐MEN has proven to have significant potential as a novel embolic material for the TACE treatment of tumors.

## 4 Experimental Section

### 4.1 Materials, Cell Lines, and Animals

Doxorubicin was purchased from Wuhan Anjie Kai Biopharmaceutical Technology Co., Ltd. (≥98%, Wuhan, China). DSPE‐PEG_2000_‐SH was purchased from Xi'an Ruixi Biotechnology Co., Ltd. (≥99%, Xi'an, China). Gallium (Ga) was purchased from a local company (Beijing, China). Trichloromethane and anhydrous methanol were purchased from Beijing Chemical Factory Co., Ltd. (Beijing, China). Dulbecco´s Modified Eagle's Medium (DMEM) and phosphate‐buffered saline (PBS) were purchased from Servicebio (China). Fetal bovine serum (FBS), penicillin/streptomycin (P/S), and trypsin were purchased from Wisent (China). Cell Counting kit‐8 was purchased from Beijing Lamblead Biotech Co., Ltd. (Beijing, China). Calcein/PI solution was purchased from Solarbio Technology Co., Ltd. (Beijing, China). Embosphere was purchased from Merit Medical Co., Ltd. (Beijing, China). VX2 cells were provided by the Cancer Laboratory of the General Hospital of the PLA (Beijing, China). The New Zealand white rabbits and the beagle dogs were purchased from Beijing Jinmuyang Experimental Animal Breeding Co., Ltd. (Beijing, China). All the animal procedures were carried out under the guidelines approved by the Institutional Animal Care and Use Committee (IACUC) of Beijing Jinmuyang Experimental Animal Breeding Co., Ltd. (permit number: jmy‐2023‐0405).

### 4.2 Preparation and Characterization of Dox‐m

The thin‐film hydration method was used to prepare doxorubicin‐loaded micelles (Dox‐m). A suitable amount of doxorubicin (Dox) and DSPE‐PEG_2000_‐SH was dissolved in an organic solvent (methanol/chloroform = 1:1, v/v). The Dox solution and DSPE‐PEG_2000_‐SH solution were mixed thoroughly at a mass ratio of 1:10 and transferred to a 50 mL round‐bottom flask. The organic solvent was removed using a rotary evaporator under vacuum (IKA RV10, Germany), and an appropriate amount of deionized water was added to dissolve the lipid film. The mixture was heated in a water bath at 60°C for 30 min, then rapidly cooled at −20°C for 4 min. Free Dox in the solution was removed by filtration through a 0.22 µm aqueous filter membrane to obtain the Dox‐m solution. Dox‐m formulations with Dox to DSPE‐PEG_2000_‐SH mass ratios of 1:5 and 1:20 were prepared using the same method. The morphology and size of Dox‐m were observed by transmission electron microscopy (TEM, Hitachi 7700, Japan). The particle size and zeta potential of Dox‐m were measured using a Malvern Zetasizer (Nano ZSP 3600, UK). The drug loading capacity (DLC) and encapsulation efficiency (EE) of Dox‐m were measured by ultracentrifugation. A certain amount of freeze‐dried Dox‐m was added to methanol and sonicated for 10 min, then centrifuged at 10 000 rpm for 10 min. The amount of Dox in the supernatant was determined using a UV–vis spectrophotometer (UV2500, China). The drug loading capacity (DLC) and encapsulation efficiency (EE) of Dox‐m were calculated using the following formulas:

$$\mathrm{DLC}(\%)=W_{loaded\;Dox}\;/W_{Dox-m}\times 100\%$$

$$\mathrm{DLE}(\%)=W_{loaded\;Dox}\;/W_{Dox\;in\;feed}\times 100\%$$

W_loaded Dox_ is the calculated amount of Dox; W_Dox‐m_ is the mass of the micelles; W_Dox in feed_ is the amount of Dox administered during the preparation of Dox‐m.

### 4.3 Preparation of X‐MEN

Liquid gallium was added dropwise into a 50 mL centrifuge tube containing a Dox‐m solution (5 mg/mL) and processed using an ultrasonic homogenizer (JY92‐IIN, China) for 5 s. Gallium microspheres loaded with Dox‐m (X‐MEN) with diameters ranging from 40 to 70 µm were isolated using a cell strainer. Similarly, liquid gallium was added to a PBS solution to obtain unloaded gallium microspheres (GaMs).

The drug loading capacity of X‐MEN was quantitatively determined using UV–vis spectrophotometry. A calibration curve was established by measuring the absorbance of Dox‐m solutions at different concentrations at 232 nm. After co‐treatment with Ga droplets, the Dox‐m solution was centrifuged at 2000 rpm for 5 min. The supernatant was carefully collected, diluted appropriately, and transferred to a cuvette for UV–vis absorbance measurement. The absorbance of the supernatant was recorded at the predetermined wavelength using a UV–vis spectrophotometer, representing the concentration of residual Dox‐m in the solution. The remaining amount of Dox‐m was calculated based on the regression equation obtained from the standard curve, and the consumed amount of Dox‐m was subsequently determined. The drug loading capacity of X‐MEN was calculated as the ratio of the consumed Dox‐m to the total amount of Ga droplets added.

The morphology and size distribution of GaMs and X‐MEN were observed using an optical microscope (LEICA DMI3000B, Germany). To confirm the presence of Dox‐m on the microsphere surface, fluorescence imaging of the GaMs and X‐MEN was performed using a confocal laser scanning microscope (CLSM, LSM710, Germany) with an excitation wavelength of 485 nm and an emission wavelength of 590 nm. Scanning electron microscopy (SEM, Hitachi SU8220, Japan) and TEM were used to characterize the microspheres, and energy‐dispersive x‐ray spectroscopy (EDS, Horiba, Japan) was employed for elemental analysis. Similar to Dox‐m, the drug loading capacity of X‐MEN was calculated using a UV–vis spectrophotometer.

### 4.4 Mechanical Property Testing of X‐MEN

The compression properties of Embosphere, GaMs, and X‐MEN were evaluated using a Microtester G2 (CellScale, Canada). Prior to testing, all samples were filtered through a 70 µm cell strainer to obtain uniformly sized microspheres. Deionized water was added to the sample chamber and heated to maintain a constant temperature of 37°C. A small amount of Embosphere was aspirated using a rubber‐bulb pipette and deposited onto the iron sample stage. Unidirectional flushing with a pipette gun was performed to isolate a single microsphere within the viewing field beneath the sample rod. The rod was then positioned above the microsphere. A cylindrical probe (diameter: 0.5588 mm) was used to compress the commercial microspheres to 20% of their original diameter. Images of the compression states, along with load, displacement, and diameter data, were recorded throughout the compression process. The same procedure was repeated to test the compression properties of GaMs and X‐MEN. The tensile properties of Embosphere, GaMs, and X‐MEN were analyzed using the Microtester G2. The samples were pre‐treated following the same protocol as in the compression test to ensure uniform size. A single Embosphere was isolated on the sample stage using the above method. A probe (diameter: 0.5588 mm) was placed above the Embosphere and compressed to 20% of its original diameter. Subsequently, the probe was vertically lifted by 100 µm to initiate tensile deformation. During the stretching process, images of the tensile state as well as data on load, displacement, and diameter were recorded. The same procedure was repeated to evaluate the stretching properties of GaMs and X‐MEN. The Young's modulus (E) was calculated using the formula:

$$E=\mathrm{FR}/\pi \;\left(2RD^{2}-d^{3}\right)$$

F represents the applied load, R is the microsphere radius, and d denotes the compression displacement.

Rheological measurements of Embosphere, GaMs, and X‐MEN were performed using a strain‐controlled MCR 302 rheometer. All measurements were conducted at 37°C. Equal volumes of Embosphere, GaMs, and X‐MEN were placed on the sample stage to measure their storage modulus (G′) and loss modulus (G′′) under different shear strains. Using the same method, their viscosity values were measured at different shear rates.

### 4.5 Drug Release of X‐MEN

A medium with a pH of 7.4 was used to simulate the normal physiological environment. A certain number of X‐MEN was placed in the release medium and dialyzed at 37°C. A certain volume of samples was taken at different time points, and the amount of Dox was determined by measuring UV absorbance. The cumulative release of Dox was then calculated based on the absorbance values at different time intervals.

### 4.6 Cytotoxicity Test

The CCK‐8 assay was used to study the toxicity of X‐MEN microspheres on VX2 cells. VX2 cells were cultured in complete medium (89% DMEM, 10% fetal bovine serum, and 1% Penicillin and streptomycin) in an incubator set at 37°C with 5% CO2. Specifically, VX2 cells were seeded in a 96‐well plate at a density of 5000 cells per well (in 100 µL of medium) and cultured for 24 h. The culture medium was then removed, and 100 µL of complete medium containing Dox‐m (32.5 µg/mL), GaMs (59 mg/mL), or X‐MEN (59 mg/mL) was added to the wells. Cells treated with PBS served as the control. After 48 h of incubation, the culture medium was removed, and the cells in the 96‐well plate were washed with PBS. Subsequently, 100 µL of complete medium containing 10% CCK‐8 reagent was added, and the cells were incubated for an additional 2 h. Cell viability was quantified by measuring the absorbance at 450 nm using a microplate reader (Thermo Fisher, USA).

VX2 cells were seeded at a density of 10 000 cells per well in confocal culture dishes. After 24 h, the culture medium was removed, and complete medium containing Dox‐m, GaMs, or X‐MEN was added to the wells. The PBS group served as the control. After 24 h of incubation, the culture medium was removed, and the cells were washed with PBS. The cells were then treated with Calcein/PI solution and washed with PBS. The fluorescence of the cells (green for live cells, red for dead cells) was observed using a confocal laser scanning microscope.

### 4.7 Blood Compatibility of X‐MEN

Blood compatibility testing was performed using rabbit whole blood treated with sodium citrate. A total of 0.5 mL each of saline, deionized water, PBS, Dox‐m (2.25 mg/mL), GaMs (5.9 g/mL), and X‐MEN (5.9 g/mL) was placed into 1.5 mL centrifuge tubes. The sodium citrate‐treated blood was diluted 50‐fold with normal saline. Then, 0.5 mL of the diluted blood was added to each centrifuge tube. The saline group served as the negative control, and the deionized water group served as the positive control. All samples were incubated in a constant‐temperature shaker at 37°C for 12 h. Afterward, the samples were centrifuged at 2000 rpm for 5 min, and the supernatant was transferred to a 96‐well plate. The absorbance at 545 nm was measured using a microplate reader. The hemolysis rate was calculated using the formula:

$$\mathrm{Hemolysis}(\%)=(A_{sample}-A_{negative})/A_{positive}\times 100\%$$

A_sample_ is the absorbance of the sample at 545 nm; A_negative_ is the absorbance of the negative control group at 545 nm; A_positive_ is the absorbance of the positive control group at 545 nm.

1 µL of PBS was placed at the bottom of the 96‐well plate along with Dox‐m (3.25 mg/mL), GaMs (5.9 g/mL), and X‐MEN (5.9 g/mL), respectively. Next, 900 µL of sodium citrate‐treated blood was mixed with 100 µL of calcium chloride (0.1 mol/mL) solution and vortexed for 10 s. Then, 50 µL of the mixture was added to the wells containing the different materials. At 1‐min intervals, the wells were washed with saline until the solution became transparent. The PBS well served as the control group.

### 4.8 In Vitro Coagulation Index Measurement

Equal volumes of PBS, Dox‐m (3.25 mg/mL), GaMs (5.9 g/mL), and X‐MEN (5.9 g/mL) were placed in 50 mL centrifuge tubes. Then, 1 mL of sodium citrate‐treated blood was added, followed by the addition of 100 µL of calcium chloride solution (0.2 mol/L) to initiate coagulation. After 5 min, 25 mL of deionized water was added to each centrifuge tube, and the samples were incubated on a constant‐temperature shaker at 37°C for 5 min. Subsequently, the supernatant was transferred to a 96‐well plate, and the absorbance at 450 nm was measured using a microplate reader. The blank group was set as the control, and its absorbance value was normalized to 100 as the reference value. The blood coagulation index (BCI) was calculated using the formula:

$$\mathrm{Coagulation}=A_{sample}\;/A_{control}\times 100$$

A_sample_ is the absorbance of the sample at 450 nm; A_control_ is the absorbance of the control group at 450 nm.

### 4.9 Safety of X‐MEN In Vivo

Safety evaluation experiments were conducted in healthy rabbits. Fifteen rabbits were randomly divided into five groups (*n* = 3 per group). After arterial puncture (using the same method as described above), each group was injected with PBS, Dox‐m, GaMs, Embosphere, or X‐MEN. Seven days post‐injection, blood tests were performed to measure hematological and biochemical parameters, including white blood cell (WBC) count, red blood cell (RBC) count, platelets (PLT), urea, serum creatinine (Scr), serum albumin (ALB), alanine aminotransferase (ALT), and aspartate aminotransferase (AST). Subsequently, all rabbits were euthanized via air embolism, and major organs, including the heart, liver, spleen, lung, and kidneys, were collected and fixed in a 4% paraformaldehyde solution for 24 h. After routine dehydration, embedding, and sectioning, H&E staining was performed to evaluate histological changes in the tissues under an optical microscope. H&E and Masson staining were performed on the embolized rabbit ears to evaluate foreign‐body reactions, inflammatory cell infiltration, and potential tissue fibrosis.

### 4.10 X‐Ray Imaging and Vascular Embolization Performance of X‐MEN

Computed tomography (CT, Germany) was used to evaluate the radiopacity of X‐MEN. Deionized water, PBS, Dox‐m, Iohexol, GaMs, and X‐MEN were placed in a 96‐well plate and subjected to CT scanning. The radiopacity of X‐MEN was analyzed by measuring the CT values. Female New Zealand White rabbits were anesthetized, and a 24‐G intravenous catheter was used to puncture the central artery of the rabbit's ear in the direction of blood flow. Through the catheter, 200 µL each of Embosphere, GaMs, and X‐MEN was respectively injected into the central arteries of different rabbits' ears. x‐ray radiography was performed before and after the procedure to observe the position of the embolic agents within the vessels. Digital subtraction angiography (DSA, Germany) was performed before and after the procedure to observe the embolization of the rabbit ear arteries in each group. The PeriCam PSI system (Perimed AB, Sweden) was used to monitor changes in the rabbit ear blood vessels over 14 days to assess the embolization effect in each group. The morphological changes in the rabbit ears were recorded for three weeks, and x‐ray imaging was performed periodically to study the effectiveness and stability of X‐MEN as an intravascular embolic agent.

### 4.11 Establishment of the Rabbit VX2 Tumor Model

The VX2 tumor tissue was minced into fragments smaller than 1 mm^3^ and suspended in a small volume of saline to facilitate injection. Female New Zealand White rabbits weighing 2–3 kg were anesthetized, and 0.1 mL of the tissue suspension was injected subcutaneously adjacent to the central auricular artery. Tumor growth was monitored regularly until the volume reached approximately 500 mm^3^.  The tumor volume was calculated using the following formula:

$$V=a\times b^{2}\;/2$$

a is the maximum diameter of the tumor; b is the minimum diameter of the tumor.

### 4.12 Treatment of the Rabbit VX2 Tumor Model

Sixteen New Zealand White rabbits bearing VX2 tumors were randomly divided into four groups (*n* = 4 per group): PBS group (control group), Dox‐m group, GaMs group, and X‐MEN group. TACE was performed when the tumor volume reached 500 mm^3^. A 24‐G intravenous catheter was used to puncture the central artery of the rabbit's ear in a retrograde direction (against the blood flow), followed by DSA examination to identify the tumor‐feeding artery and adjust the catheter position. PBS, Dox‐m (0.325 mg), GaMs (590 mg), or X‐MEN (590 mg) were injected through the catheter under DSA guidance for treatment, respectively. x‐ray and CT scans were performed before and after treatment, and laser speckle contrast imaging was used to monitor changes in the rabbit ear blood vessels over 7 days. The embolic effect and radiopacity of X‐MEN in tumor vessels were evaluated by comparison with Embosphere. Tumors were observed every two days to plot tumor volume change curves. One rabbit from each group was randomly euthanized on day 7, and tumor tissues were harvested for H&E, Ki‐67, and TUNEL staining.

### 4.13 The Long‐Term Embolization Effect Assessment in the Beagle Dog Model

Beagle dogs were anesthetized, and their necks were shaved. After disinfection, a transverse incision of approximately 1 cm was made at the common carotid artery, and the subcutaneous tissue and muscles were dissected layer by layer to expose the right common carotid artery. The distal segment of the right common carotid artery was ligated. A 4F catheter sheath was inserted centripetally into the common carotid artery using the Seldinger technique. Under DSA guidance, a 4F vertebral artery catheter was advanced through the sheath into the abdominal aorta to perform left internal iliac artery angiography. The catheter position and direction were adjusted to super‐selectively target the left prostatic artery branch. A 2.4‐F microcatheter was then super‐selected into the left prostatic artery, and contrast agent was injected to confirm prostate staining. The catheter's tip position was confirmed to be optimal, showing only prostate staining without visualization of other collateral arteries. After ensuring no contrast reflux, embolization treatment was initiated. Under DSA, GaMs was injected into the left prostatic artery through the catheter. The embolization process was dynamically observed under fluoroscopy and stopped once blood flow ceased, with careful monitoring for any reflux to avoid inadvertent embolization of other vessels. After embolization, another angiogram was performed to confirm complete embolization of the prostate artery. The same procedure was then applied to the right prostate artery. After embolization, all catheters and sheaths were withdrawn, and the puncture site was pressed for 20 min, followed by wound suturing. Pre‐ and post‐operative CT, MRI scans, and blood tests were performed. Collect dog samples including prostate, heart, liver, spleen, lung, kidney, brain, bladder, rectum, ureter, fore and hind limb, feces, and urine. Weigh the samples in a beaker using an analytical balance, add 10 mL of nitric acid solution, continue heating and evaporating until it becomes nearly viscous, then add 5 mL of deionized water and continue heating. When it evaporates to near dryness, remove and cool it down. Rinse the beaker with deionized water solution, dilute to 10 mL with deionized water, shake well, and proceed with ICP‐MS analysis.

### 4.14 Analysis of Macrophage Polarization by Flow Cytometry

RAW 264.7 cells were seeded in 6‐well plates at a density of 2 × 10^5^ cells per well and cultured overnight. The cells were then incubated with PBS (Control), Embosphere, GaMs, or X‐MEN microspheres for 24 h. Following treatment, the cells were harvested by gentle scraping or trypsinization and washed twice with cold PBS containing 1% BSA. To block non‐specific binding, cells were incubated with an anti‐CD16/32 antibody (Fc block) for 10 min at 4°C. Subsequently, the cells were stained with fluorochrome‐conjugated antibodies targeting macrophage surface markers. Specifically, anti‐CD86 antibody and anti‐CD80 antibody were used to identify the M1 phenotype to evaluate the immunogenicity of X‐MEN and GaMs. Samples were analyzed using flow cytometry (Cytek Aurora). Anti‐mouse CD80 (clone 16‐10A1, catalogue number 12‐0801‐81, 1:100 dilution) was purchased from Introvergen. Anti‐mouse CD86 (clone GL1, catalogue number 747436, 1:100 dilution) was purchased from BD.

### 4.15 Cytokine Quantification by ELISA

For in vitro studies, RAW 264.7 cells were seeded in 6‐well plates and cultured until they reached 70%–80% confluence. The cells were then incubated with PBS (Control), Embosphere, GaMs, or X‐MEN microspheres for 24 h. Subsequently, the cell culture supernatants were harvested and centrifuged at 1000×g for 10 min at 4°C to remove cellular debris. The supernatants were stored at −80°C until analysis. For in vivo studies, C57BL/6 mice were randomly divided into four groups (*n* = 5) and treated with PBS, Embosphere, GaMs, or X‐MEN. Twenty‐four hours post‐administration, peripheral blood samples were collected. Serum was separated by centrifugation at 3000 rpm for 15 min. The concentrations of proinflammatory cytokines (IL‐6, TNF‐α, IL‐2) were quantified using commercial Enzyme‐Linked Immunosorbent Assay (ELISA) kits (DAKEWEI, catalogue numbers 1210602, 1217202, 1210202) according to the manufacturer's instructions. The optical density (OD) was measured at 450 nm using a microplate reader (Infinite 200 PRO, TECAN, Switzerland).

### 4.16 Hematological Analysis

C57BL/6 mice were randomly assigned to four groups (*n* = 5) and treated with PBS (Control), Embosphere, GaMs, or X‐MEN. Twenty‐four hours post‐administration, peripheral blood samples were collected into tubes containing EDTA‐K2 as an anticoagulant to prevent clotting. Whole blood samples were immediately analyzed using an automated hematology analyzer. Key hematological parameters were quantified, including total white blood cell count (WBC), lymphocyte count (Lymph), monocyte count (Mon), granulocyte count (Gran), and platelet count (PLT).

### 4.17 Statistical Analysis

Data analysis in this study was performed using GraphPad Prism and Origin software. Quantitative data are expressed as mean ± standard error of the mean (Mean ± SEM). The sample size (n) is indicated in the figure legend. Data are presented as means ± SEM, *n* = 3. A *p*‐value of < 0.05 was considered statistically significant. The specific notation is as follows: ns *p* > 0.05, ^*^ *p* < 0.05, ^**^ *p* < 0.01, ^***^ *p* < 0.001, ^****^ *p* < 0.0001.

## Conflicts of Interest

The authors declare no conflicts of interest.

## Supporting information




**Supporting File 1**: advs74849‐sup‐0001‐SuppMat.docx.

**Supporting File 2**: advs74849‐sup‐0002‐MovieS1.avi.

**Supporting File 3**: advs74849‐sup‐0003‐MovieS2.avi.

**Supporting File 4**: advs74849‐sup‐0004‐MovieS3.mp4.

## Data Availability

The data that support the findings of this study are available in the supplementary material of this article.
