# Supplementary material for: Novel Vascular‐Adaptive Liquid Metal Microspheres Enable Visualized Arterial Embolization Therapy
Source: Adv Sci (Weinh). 2026 Mar 20;13(30):e21441. doi: 10.1002/advs.202521441 (PMC13248812; doi:10.1002/advs.202521441)
Supplement: Supplementary file 1 — Supporting File 1: advs74849‐sup‐0001‐SuppMat.docx. [file ADVS-13-e21441-s001.docx]

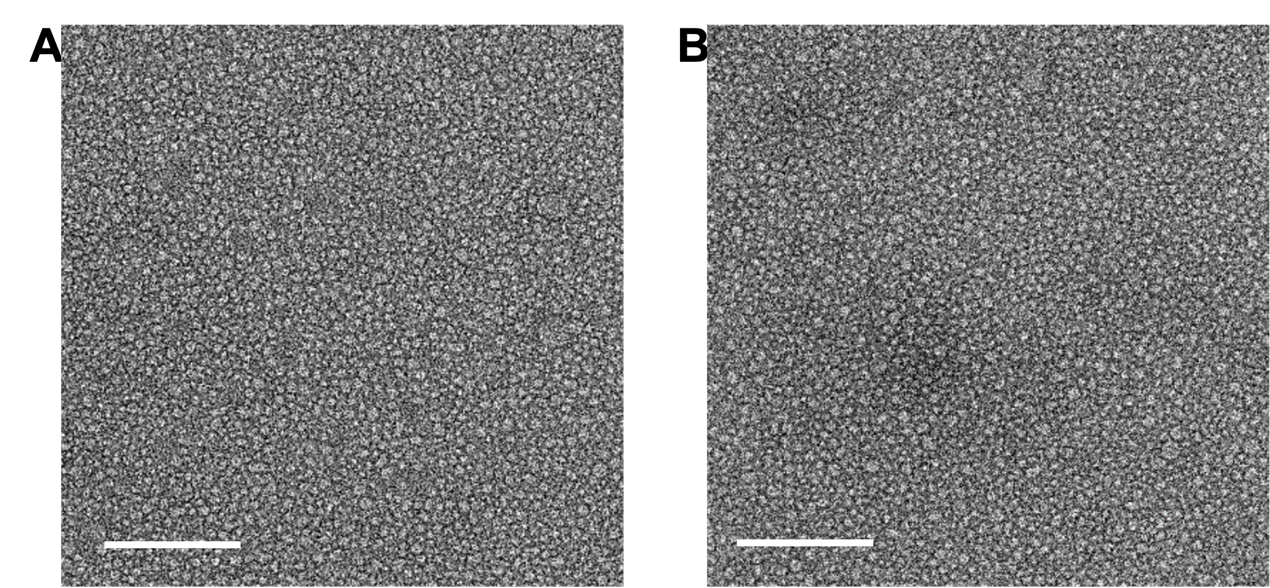


**Figure S1. TEM images of Dox-m prepared with a Dox to DSPE-PEG2000-SH ratio of 1:5 (A) and 1:20 (B). Scale bar: 200 nm.**


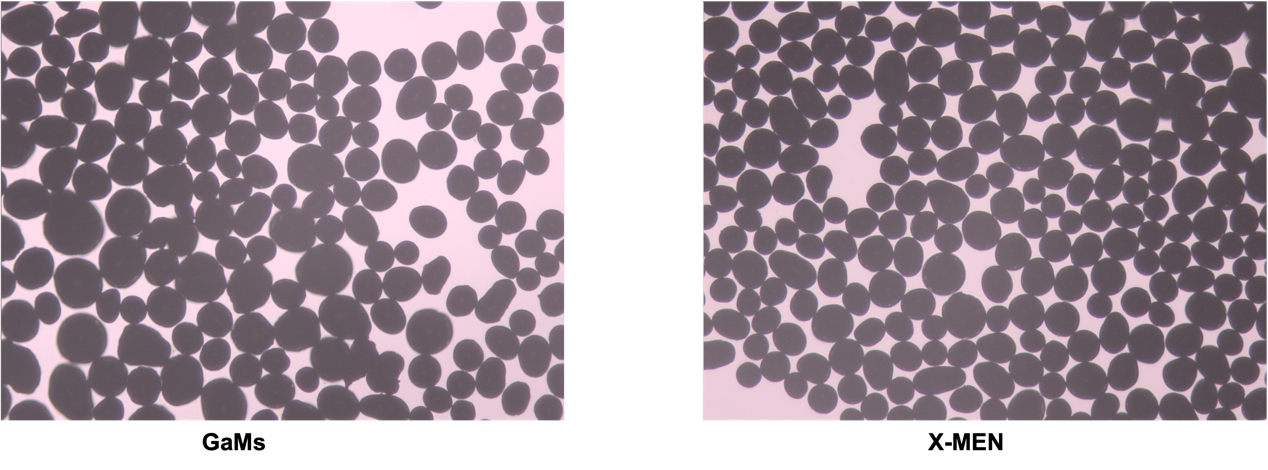


**Figure S2. Optical microscope images of GaMs and X-MEN.**

**
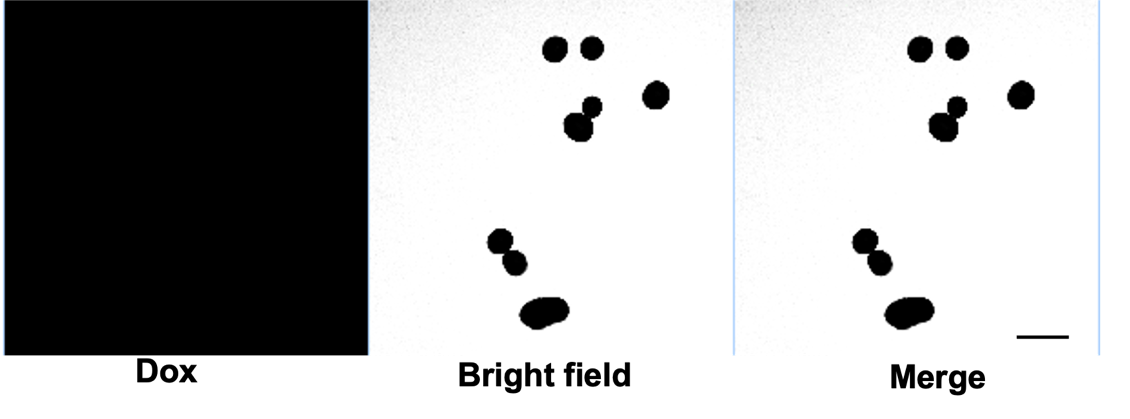
**

**Figure S3. Confocal microscope image of GaMs. Scale bar: 100 µm.**


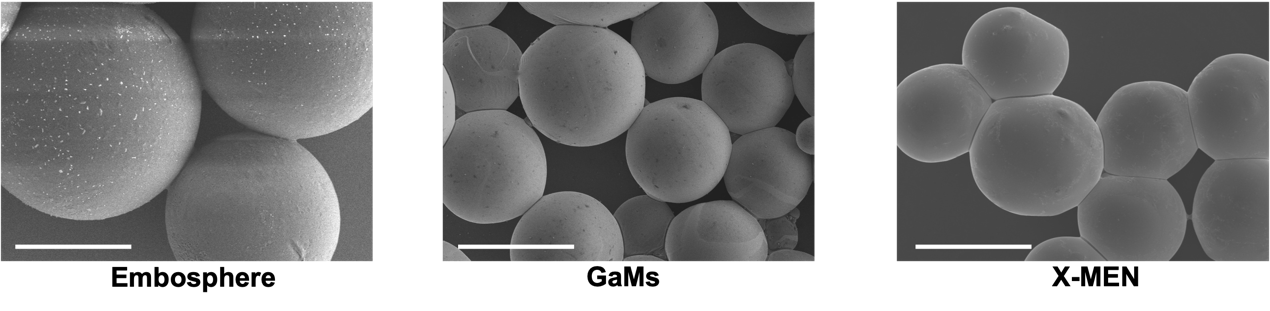


**Figure S4. SEM images of Embosphere, GaMs, and X-MEN. Scale bar: 50 µm.**

**
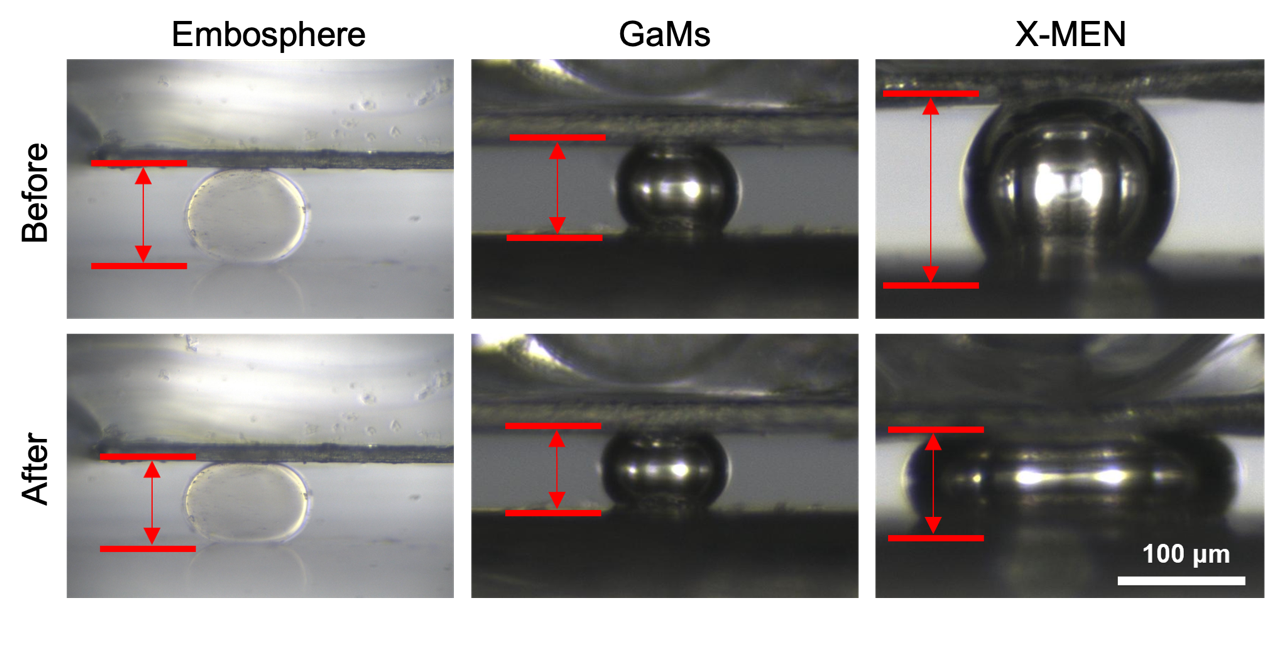
**

**Figure S5. Maximum compression ratio tests of Embosphere, GaMs, and X-MEN.**


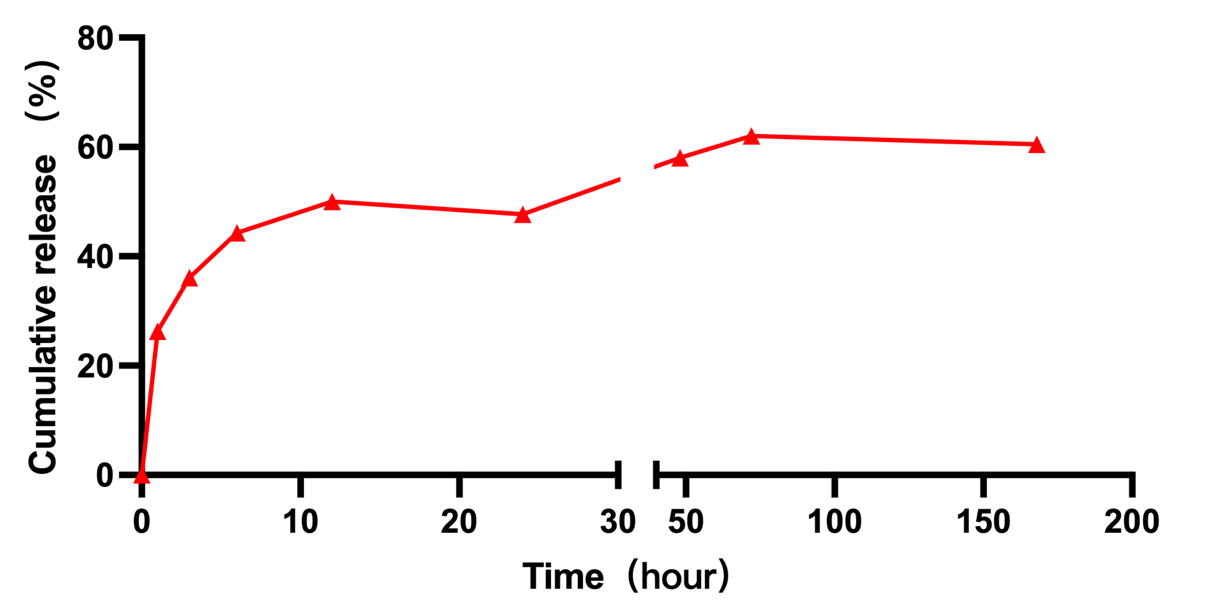


**Figure S6. Cumulative Dox release from X-MEN in PBS at PH 7.4 over 7 days.**


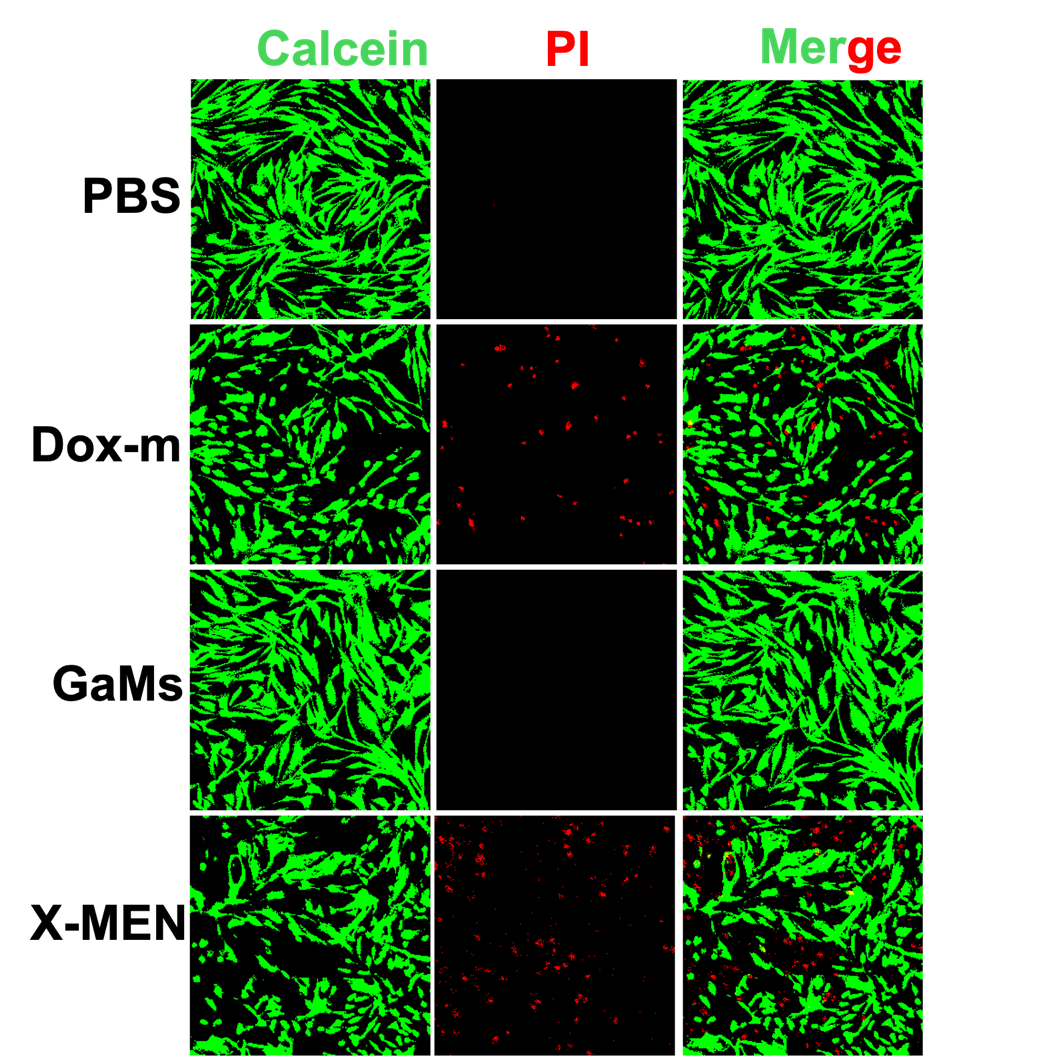


**Figure S7. Calcien/PI staining of VX2 cells after 24 hours of co-culture with PBS, Dox-m, GaMs and X-MEN** **(half concentration).**


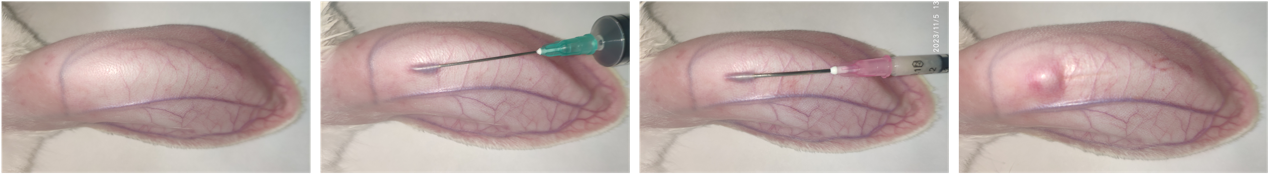


**Figure S8. Establishment of the VX2 tumor model.**


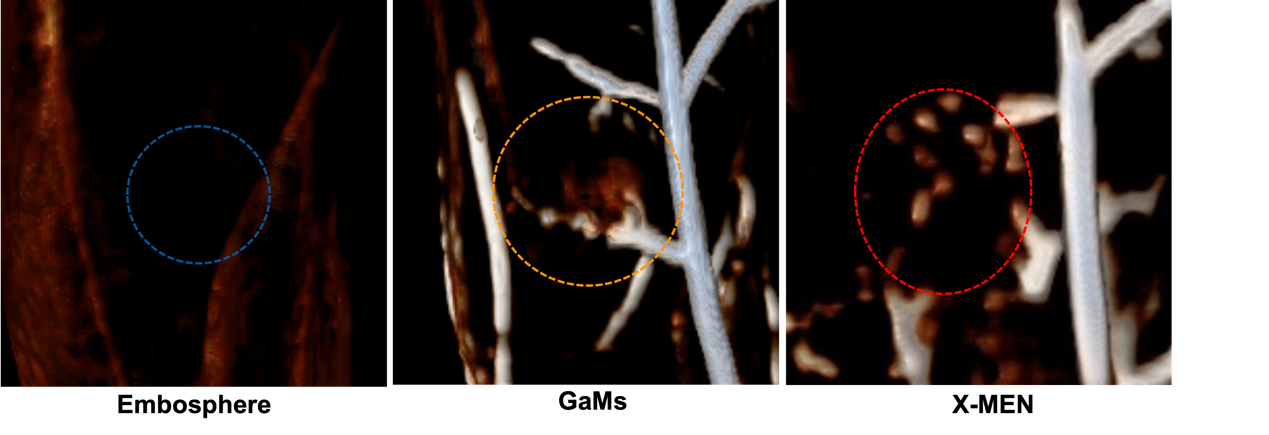


**Figure S9. 3D-CT images of tumor after embolization with** **Embosphere, GaMs, and X-MEN.**


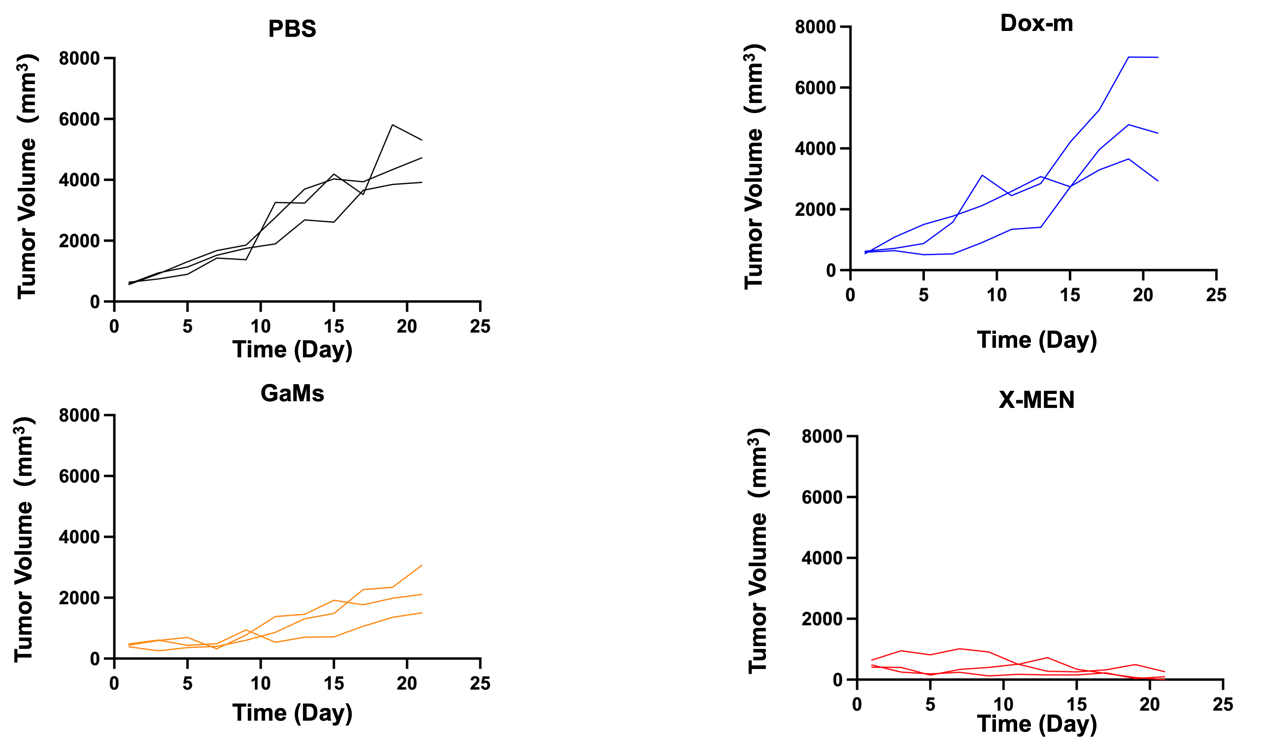


**Figure S10. Tumor volume changes in each group.**

**
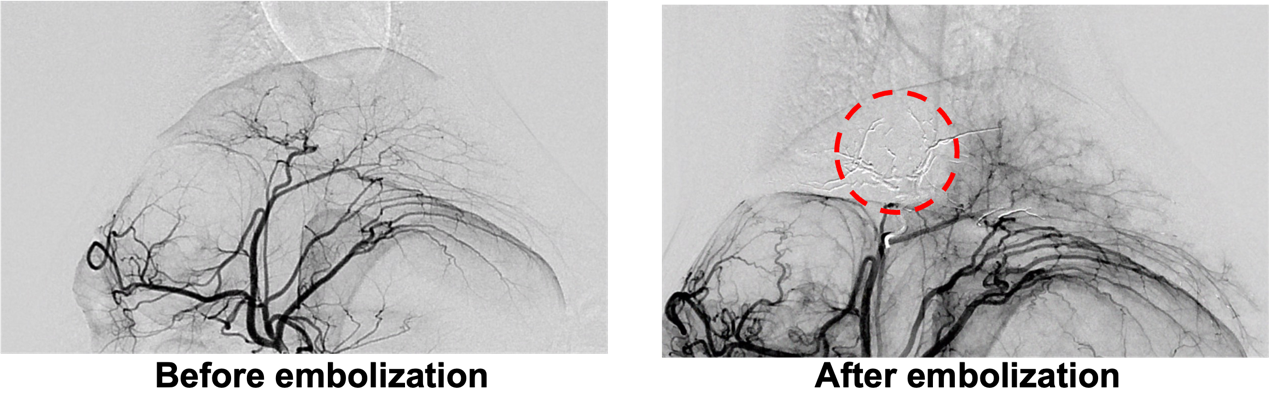
**

**Figure S11. Images from digitally subtracted angiography (DSA) of the tumor before embolization and after embolization. DSA before embolization can help identify the tumor location (circle markers) and vascular anatomy. DSA after embolization confirms the disappearance of tumor staining (circle markers).**

**
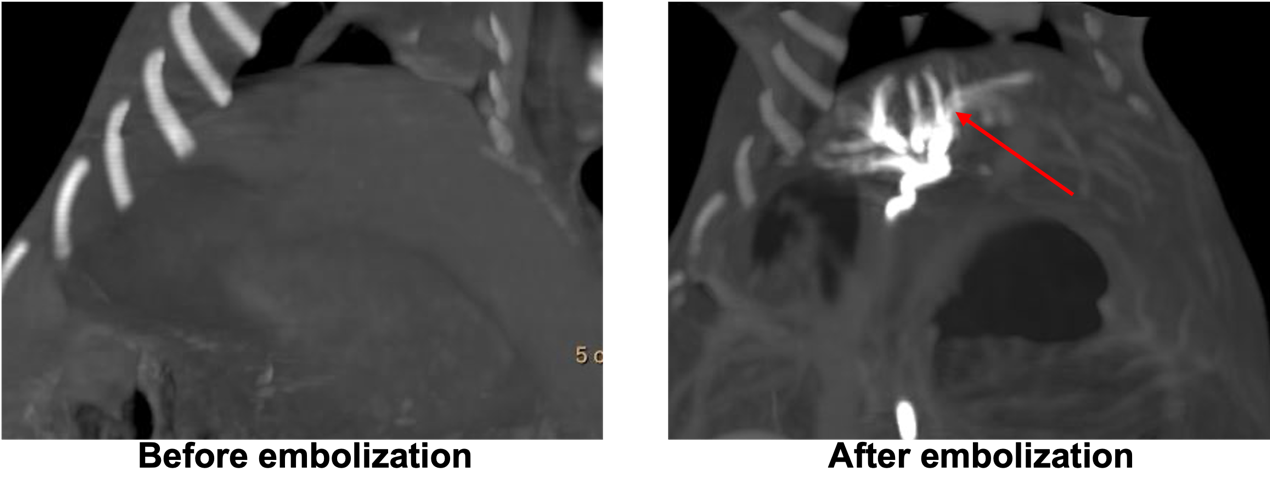
**

**Figure S12. CT images of tumor before and after embolization. The image after embolization shows the location of the microspheres (arrow heads).**


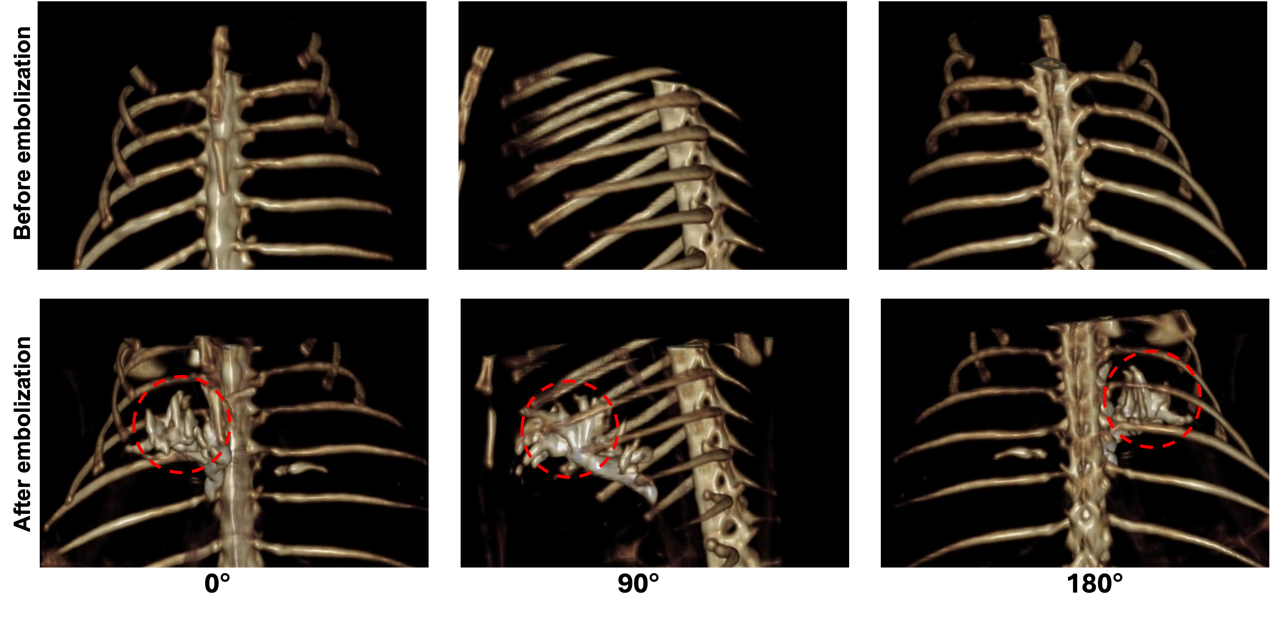


**Figure S13. 3D-CT images of tumor before and after embolization. The images after embolization show the location of the microspheres (circle markers) from different angles.**

**
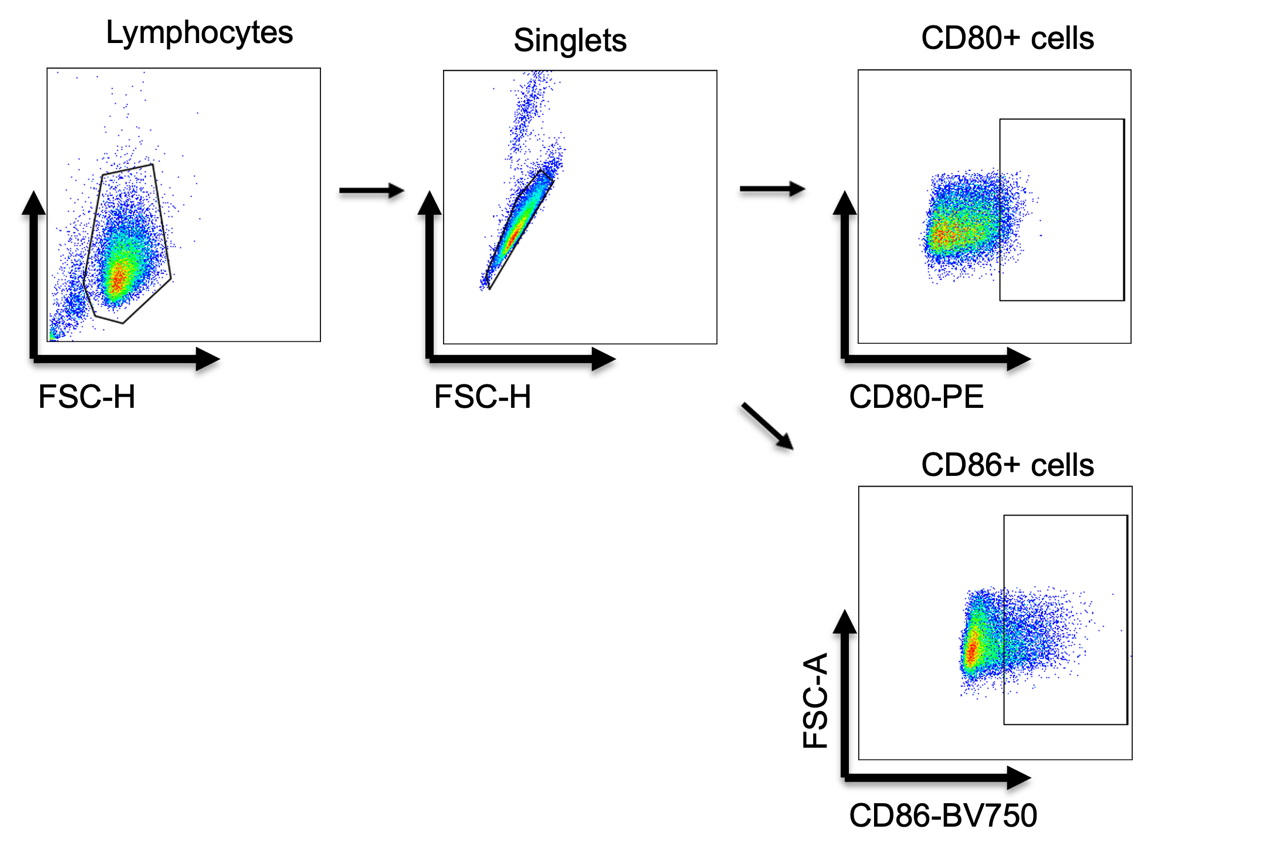
**

**Figure S14. The flow-cytometry gating strategy.**

**
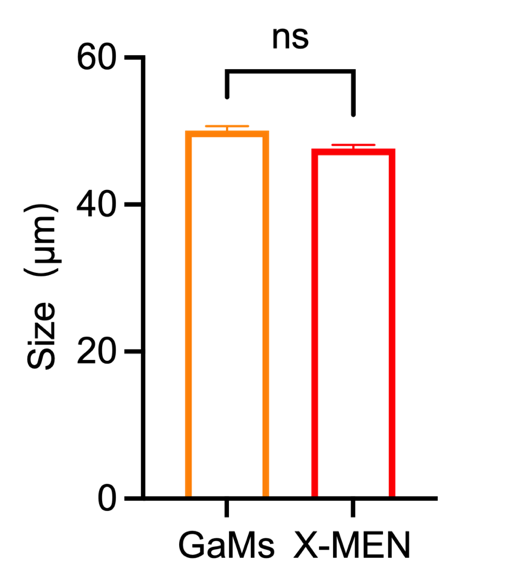
**

**Figure S15. Particle sizes of GaMs and X-MEN**
